# Combined proton–photon therapy for non‐small cell lung cancer

**DOI:** 10.1002/mp.15715

**Published:** 2022-05-25

**Authors:** Florian Amstutz, Silvia Fabiano, Louise Marc, Damien Charles Weber, Antony John Lomax, Jan Unkelbach, Ye Zhang

**Affiliations:** ^1^ Center for Proton Therapy Paul Scherrer Institute Villigen Switzerland; ^2^ Department of Physics ETH Zurich Zurich Switzerland; ^3^ Department of Radiation Oncology University Hospital Zurich Zurich Switzerland; ^4^ Department of Radiation Oncology University Hospital Bern Bern Switzerland

**Keywords:** combined proton‐photon therapy, lung cancer, photon therapy, proton therapy

## Abstract

**Purpose:**

Advanced non‐small cell lung cancer (NSCLC) is still a challenging indication for conventional photon radiotherapy. Proton therapy has the potential to improve outcomes, but proton treatment slots remain a limited resource despite an increasing number of proton therapy facilities. This work investigates the potential benefits of optimally combined proton–photon therapy delivered using a fixed horizontal proton beam line in combination with a photon Linac, which could increase accessibility to proton therapy for such a patient cohort.

**Materials and methods:**

A treatment planning study has been conducted on a patient cohort of seven advanced NSCLC patients. Each patient had a planning computed tomography scan (CT) and multiple repeated CTs from three different days and for different breath‐holds on each day. Treatment plans for combined proton–photon therapy (CPPT) were calculated for individual patients by optimizing the combined cumulative dose on the initial planning CT only (non‐adapted) as well as on each daily CT respectively (adapted). The impact of inter‐fractional changes and/or breath‐hold variability was then assessed on the repeat breath‐hold CTs. Results were compared to plans for IMRT or IMPT alone, as well as against combined treatments assuming a proton gantry. Plan quality was assessed in terms of dosimetric, robustness and NTCP metrics.

**Results:**

Combined treatment plans improved plan quality compared to IMRT treatments, especially in regard to reductions of low and medium doses to organs at risk (OARs), which translated into lower NTCP estimates for three side effects. For most patients, combined treatments achieved results close to IMPT‐only plans. Inter‐fractional changes impact mainly the target coverage of combined and IMPT treatments, while OARs doses were less affected by these changes. With plan adaptation however, target coverage of combined treatments remained high even when taking variability between breath‐holds into account.

**Conclusions:**

Optimally combined proton‐photon plans improve treatment plan quality compared to IMRT only, potentially reducing the risk of toxicity while also allowing to potentially increase accessibility to proton therapy for NSCLC patients.

## 1 INTRODUCTION

Lung cancer is the second most diagnosed cancer worldwide. With an estimated 2.2 million new cases in 2020, lung cancer is only slightly surpassed by female breast cancer (2.3 million) and is the leading cause of cancer deaths, contributing to 18% of all cancer‐related mortalities.^1^ The majority (83%) of diagnosed lung cancers are non‐small cell lung cancers (NSCLC), whose 5‐year relative survival rate across all stages is only 23%.^2^ As the majority of NSCLC patients are diagnosed at stage III or IV, radiotherapy is a compulsory treatment for most of these patients.^2^ Although the outcome of radiotherapy has been improved with the implementation of state‐of‐the‐art delivery techniques, such as intensity‐modulated radiation therapy (IMRT) and image‐guided radiotherapy, the management of NSCLC remains challenging.^3^ Consequently, multiple studies have suggested using proton therapy to further improve radiotherapy treatment outcomes for these patients.^3^, ^4^, ^5^, ^6^, ^7^, ^8^, ^9^, ^10^ Indeed, as proton therapy can substantially reduce the dose to normal tissues (e.g. heart, lung, spinal cord and the esophagus), it could lead to fewer toxicities and/or higher chance of tumor control due to the potential of dose escalation,^4^ particularly for large central tumors close to organs at risk (OARs).^3^ Unfortunately, proton therapy is still a limited resource due to the high price for installation, treatment and maintenance.^11^ Even though the number of proton centers has rapidly increased, there is still a large imbalance between the ≈ 12′000 photon treatment units and ≈ 240 proton treatment rooms.^12^, ^13^, ^14^

One possibility to increase the access to proton therapy for the NSCLC patient population could be by combining proton and photon treatments.^12^, ^15^, ^16^, ^17^ Additionally, there are challenges for a pure proton therapy for NSCLC patients such as plan robustness and motion mitigation, for which a combined proton–photon therapy (CPPT) might also have beneficial aspects. Combining proton and photon therapy is an approach that has already been shown to be of some promise for other indications.^12^, ^15^, ^16^, ^17^, ^18^, ^19^, ^20^, ^21^, ^22^, ^23^, ^24^, ^25^

Early clinical studies have investigated the combination of proton and photon therapy for tumors of the axial skeleton such as chordomas, chondrosarcomas or osteo‐ and chondroblastomas. The rationale for using combined treatments for these tumors however was due to dose‐limiting factors of conventional photon therapy at the time.^16^, ^17^, ^20^ Combined treatments have also been used to reduce skin dose by adding photon components to passively scattered proton treatments. Compared to conventional photon therapy, delivery of higher doses to the tumor while respecting constraints for the OARs was possible^20^ and outcomes and toxicities for the combined treatments were comparable to proton only treatments.^16^, ^17^ Additionally, for atypical and malignant meningiomas, combined protons and photons have shown results comparable to the best reported overall survival (OS) at that time^26^ and for spinal sarcoma treatments^21^, ^22^ in which likewise, outcomes were better than for photon only treatments and comparable to proton only treatments.^27^ Such studies indicate the clinical potential of combined treatment regimes.

More recently, a growing number of treatment planning studies and methodology‐oriented papers have once again focused on the combination of protons and photons. In,^28^ limited proton treatment slots for head and neck (HN) patients was the rationale for investigating possible combinations with photons, with the proton potentially only being used for the boost, thus reducing dose degradation as a result of anatomical changes through the treatment. This dataset of 45 head and neck cancer patients was then further investigated in,^18^ where the authors develop methods to optimally distribute a limited number of proton therapy slots over a HN patient cohort with the goal of maximizing the benefit of proton resources on the population level. In the context of liver radiotherapy, ten Eikelder et al.^23^ developed methods to determine the optimal number of fractions and dose per fraction for protons and photons based on biologically effective dose (BED), showing that combined treatments can be optimal if both modalities have complementary advantages. Other works have extended the treatment planning methodology towards jointly optimizing proton and photon dose distributions rather than planning each modality separately. Unkelbach et al.^15^ and Fabiano et al.^19^ demonstrated that, by jointly optimizing proton and photon fractions based on BED, combined treatments may be obtained that better exploit limited proton fractions by delivering an overproportionate dose with protons. Gao^24^ proposed joint optimization of IMRT and intensity‐modulated proton therapy (IMPT) plans based on their cumulative dose distribution, while adding additional objectives to enforce that protons and photons each deliver homogeneous doses to the target volume. Fabiano et al.^12^ considered joint optimization of photon and proton beams for HN patients assuming a treatment room containing a photon Linac and a fixed beam line for protons, allowing for a delivery of protons and photons in the same fraction.

Extending on this previous work,^12^ the objective here is to investigate the potential clinical benefit of adding a fixed horizontal proton beam line (FHB) to a conventional photon gantry for treating NSCLC. Such a treatment room design would have the chance to lower the costs and complexity for clinical CPPT implementation, as it would make the large and expensive proton gantry redundant. CPPT is compared to IMPT only plans from the aspects of plan quality, robustness and biological side effects, as well as in the presence of daily anatomic changes and inter‐breath hold variability. However, as an additional comparison, the CPPT scenario of using a full proton gantry is also explored.

As such, we particularly wish to study the following three questions:

1. Is there a benefit for CPPT with a FHB compared to IMRT‐only treatments?
2. Would the quality of CPPT treatments be improved if the proton component was delivered on a proton gantry?
3. Can CPPT compete with IMPT‐only treatments?

Furthermore, since substantial anatomical changes often occur for NSCLC patients between the planning and delivery phase,^29^ the above questions will be investigated in the context of both non‐adaptive and adaptive treatment strategies, and the necessity of range robust optimization is evaluated. To the best of our knowledge, this is the first investigation of the potential benefit of optimally CPPT for NSCLC patients, and is also the first work to investigate CPPT performance taking into account inter‐fractional anatomical changes.

## 2 MATERIALS AND METHODS

### 2.1 Patient cohort

**FIGURE 1:**
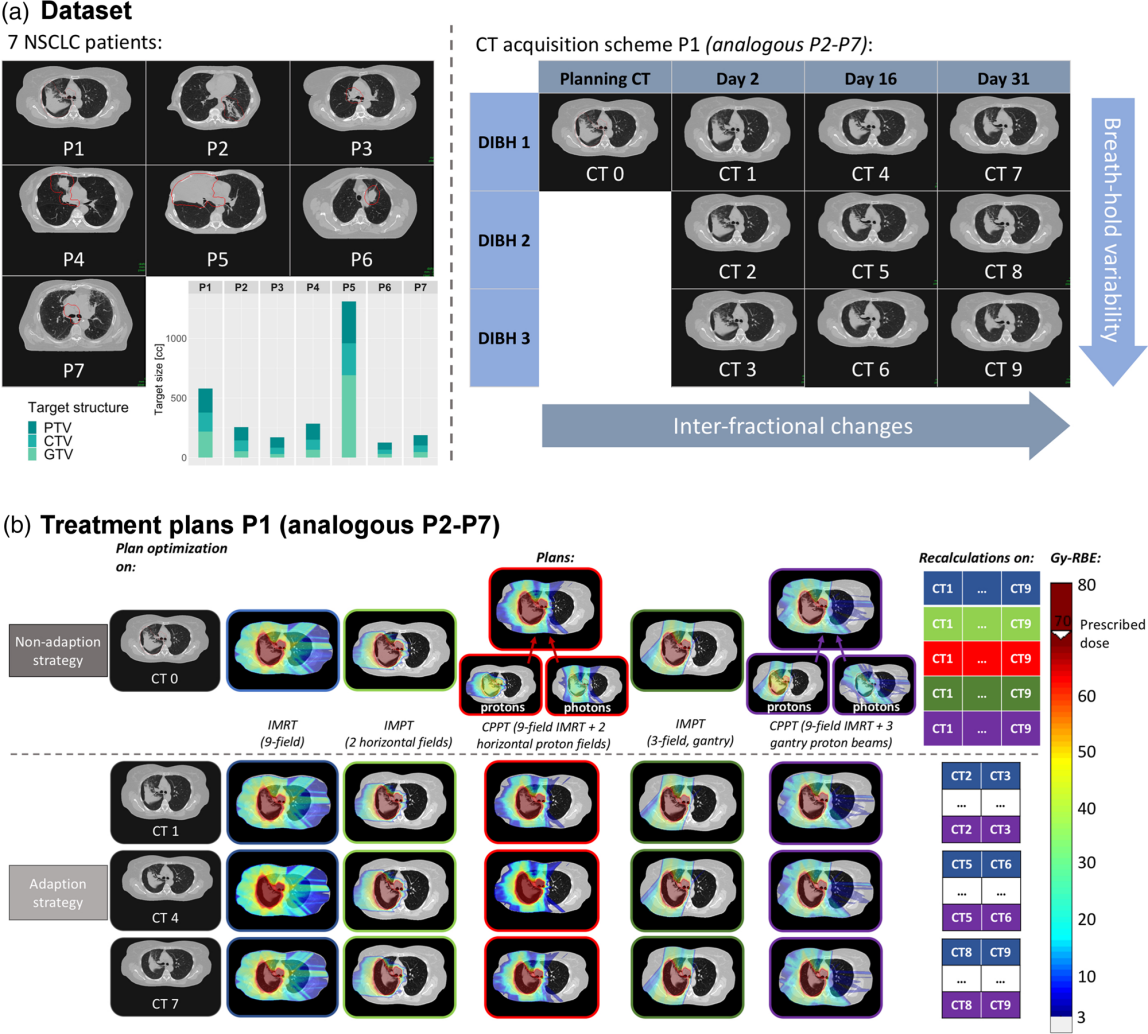
(a) Overview of the dataset including an example slice for each of the seven patients together with the target structure sizes for the different patients. The CT acquisition scheme is shown on the example of patient 1. (b) Optimized treatment plans for patient 1 together with the recalculation scheme for the non‐adaptive and the adaptive regime

Seven locally advanced NSCLC patients have been included in this retrospective treatment planning study.^30^, ^31^ The location of the tumors together with the target structure sizes are shown in Figure 1a. For each patient, one planning CT and nine repeat CTs were acquired in visually guided, voluntary deep inspiration breath‐hold (DIBH) on treatment days 2, 16, and 31. For each of these days, three CTs in different breath‐holds were acquired as shown in Figure 1a. All repeat CT's have been rigidly registered to the corresponding planning CT.

### 2.2 Treatment planning

#### 2.2.1 Treatment plan optimization

As reference plans, the following, conventional IMRT and IMPT plans were optimized:

- IMRT: 9‐field IMRT plan consisting of equispaced coplanar beams;
- Gantry IMPT: 3‐field IMPT plan with patient‐specific fields delivered by a gantry. Field angles from previously published work on these patients were used and are summarized in Table A1
- FHB IMPT: 2‐field IMPT plan delivered by a FHB at 270°, if the tumor is located on the same side as the beam line, couch angles of 20° and 340° are used, otherwise 160° and 200°

CPPT plans were then calculated by simultaneously optimizing the cumulative dose of IMRT and IMPT fields as described in ref.,^12^ resulting in non‐homogenous dose distributions from both modalities. Two different CPPT plans were created:

- FHB CPPT: CPPT plan, which optimally combines the 9 photon fields with the two proton fields.
- Gantry CPPT: CPPT combining the 9‐fields IMRT and the respective proton gantry fields.

As described above, in CPPT the proton and photon doses would be delivered consecutively in the same treatment fraction, by delivering the proton dose by the FHB, respectively gantry, followed directly by the delivery of the photon dose by the photon linac, or vice versa.

All plans were calculated to deliver 70 Gy‐RBE to the planning target volume (PTV) with optimization objectives being the same for all plans and patients. For the OARs, the mean dose to the healthy lungs (both lungs without gross tumor volume (GTV)) was minimized, for the esophagus and the spinal cord the maximum dose was optimized to be lower than 74 Gy‐RBE, respectively 45 Gy‐RBE. Additional objectives were used to optimize the sparing of the remaining healthy tissue and for plan conformity. The full optimization methodology can be found in the supplement.^32^, ^33^, ^34^ All optimized plans, with the exception of two, fulfilled the following dosimetric compliance criteria of the RTOG 1308 trial,^35^ which were not explicitly included in the objective function:

- PTV
  ○ *D*_95%_ ≥ 95%
  ○ Min dose ≥ 85%
  ○ Max dose ≤ 125%
- Heart
  ○ V30_Gy‐RBE_ ≤ 50%
  ○ V45_Gy‐RBE_ ≤ 35%
- Healthy lungs (both lungs without GTV)
  ○ V5_Gy‐RBE_ ≤ 65%
  ○ V20_Gy‐RBE_ ≤ 37%
  ○ Mean dose < 20 Gy‐RBE

The IMRT plan for the very large tumor case of patient 5 could not fulfill the *V*_20 Gy‐RBE_ and the *V*_5 Gy‐RBE_ criteria for the healthy lungs. Additionally, the FHB IMPT plan for patient 7 slightly exceeded the maximum dose criteria of the PTV.

#### 2.2.2 Baseline, non‐adaptive and adaptive plans

In the *baseline* regime, only doses optimized on the original planning CT were considered, thus ignoring any anatomic variations during the course of therapy. In contrast, for the *non‐adaptive* regime, the plan originally optimized on the planning CT was recalculated on all nine repeated CTs to investigate the effect of anatomical changes combined with inter‐breath‐hold variability on the dose distribution. Finally, for the *adaptive* regime, plans were *reoptimized* on one repeat CT of treatment days 2, 16, and 31 (CT1, CT4, CT7), and then *recalculated* on the two‐remaining repeated CTs of each day (e.g., CT2 and CT3 for CT1). With this approach, the effect of the breath‐hold variability on the adapted plans could be investigated. Thus, the importance or insignificance of adaption for IMRT, IMPT, and CPPT scenarios could be studied. A schematic representation of the two strategies with the different plans for example patient 1 can be seen in Figure 1b.

#### 2.2.3 Range robust optimization

All the previously described planning strategies have additionally been investigated in combination with range robust optimization. For this, different uncertainty scenarios of ± 3%, ± 5%, and ± 7% hounsfield unit (HU) scaling were considered. CPPT with its photon component could be more robust. Therefore, three different values were used to investigate if range robust optimization in combination with CPPT is able to mitigate anatomical changes to some extent and if yes, at which values. For this probabilistic range robust optimization, the nominal scenario was weighted with 50%, while the over‐ and undershoot scenario were weighted 25% each. To be consistent, range robust optimization was performed for all calculated plans, including IMRT plans. Setup uncertainties were not included however, these being dealt with using a conventional PTV approach.^33^

### 2.3 Treatment plan evaluations

After calculation, all treatment plans were visually evaluated, and dose–volume histograms (DVHs), as well as different dose parameter values, assessed. For the OARs, namely the healthy lungs, heart, esophagus, and spinal cord, dose parameters were chosen based on RTOG 1308. For the PTV, *D*_95%_ was used as an indicator for target coverage.

#### 2.3.1 Normal tissue complication probability

To have a more clinical assessment of treatment plan quality, three normal tissue complication probabilities (NTCPs) have been calculated to quantify the probability of toxicities for the different treatment modalities. For the lung, the risk of radiation pneumonitis was calculated as follows:

(1) $$\mathrm{NTC}P_{\mathrm{Radiation}\;\mathrm{Pneumonitis}}=\Phi \left(\frac{1.25*\mathrm{MLD}-TD_{50}}{m*TD_{50}}\right)$$

with Φ representing the cumulative distribution function of the standard normal distribution and the model parameters MLD mean lung dose, *m* = 0.45; and TD_50_ = 31.4 Gy.^36^

Additionally, two NTCP models from the Dutch national proton therapy indication protocol for lung carcinomas were used.^37^ One model for esophageal toxicity with an endpoint of a toxicity ≥ grade 2 given by

(2a) $$\mathrm{NTC}P_{\mathrm{esophageal}\;\mathrm{toxicity}>\mathrm{grade}\;2}=\frac{1}{1+e^{-S_{E}}},$$

(2b) $$\begin{array}{c} S_{E}=-3.634+1.496*\ln\left(\mathrm{MED}\right)-0.0297*\mathrm{interval}, \end{array}$$

with MED being the mean esophagus dose and interval the overall treatment time in days.

Furthermore, an NTCP model predicting the 2‐year mortality:

(3a) $$\mathrm{NTC}P_{2-\mathrm{year}\;\mathrm{mortality}}=\frac{1}{1+e^{-S_{H}}},$$

(3b) $$\begin{array}{c} S_{H}=-1.3409+0.0590*\sqrt{\mathrm{GTV}}+0.2635*\sqrt{\mathrm{MHD}}, \end{array}$$

with GTV being the volume of the gross tumor in cm^3^ and MHD the mean heart dose. All NTCPs were calculated for baseline, non‐adaptive, and adaptive regimes.

## 3 RESULTS

### 3.1 Baseline regime

**FIGURE 2:**
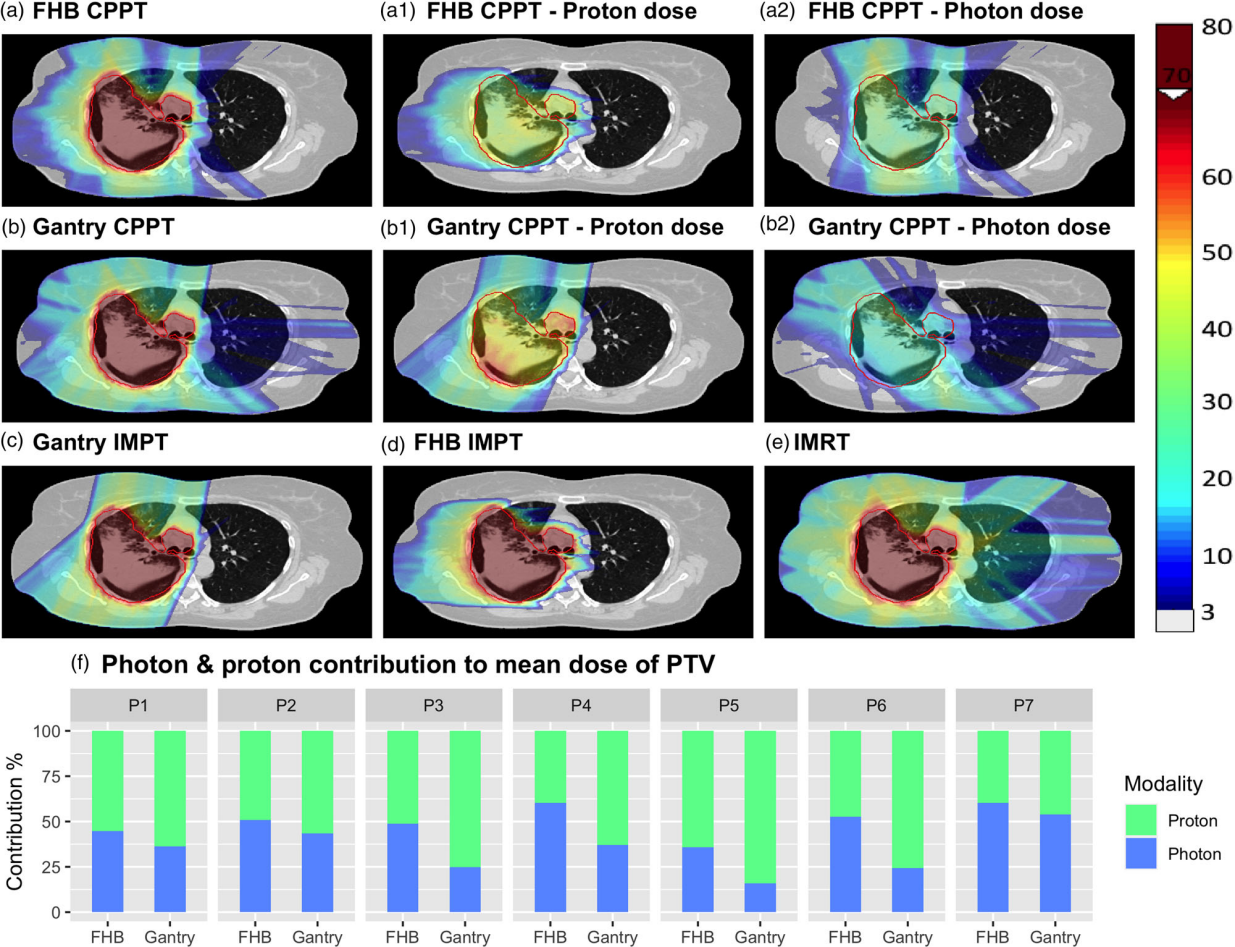
Treatment plans for patient 1 (PTV in red): (a) fixed horizontal proton beam line (FHB) combined proton–photon therapy (CPPT); (a1) proton contribution of CPPT FHB delivered by a FHB; (a2) photon contribution of FHB CPPT delivered by 9 equispaced intensity‐modulated radiation therapy (IMRT) fields; (b) gantry CPPT; (b1) proton contribution delivered by a gantry; (b2) photon contribution of the gantry CPPT plan delivered by 9 equispaced IMRT fields; (c) gantry IMPT; FHB IMPT; (e) IMRT. (f) Relative photon and proton contribution to the mean dose of the PTV for the combined proton–photon therapy with the FHB and the Gantry approach for all patients

**FIGURE 3:**
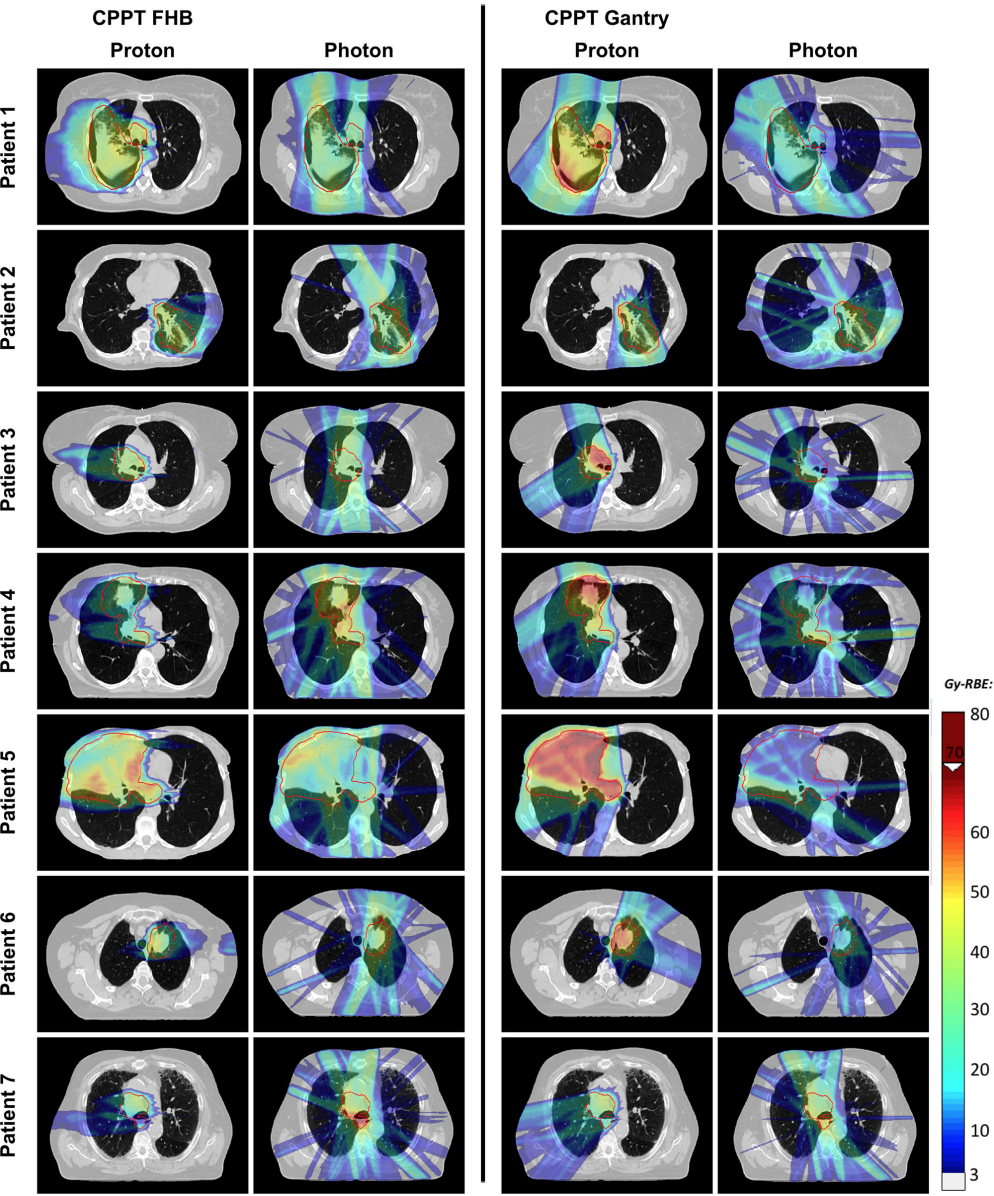
Proton and photon relative spatial dose distribution for combined proton–photon therapy (CPPT) with the fixed horizontal proton beam line (FHB) and the gantry approach for all patients

For the optimized plans, visual inspection of the doses for all patients showed a clear reduction of the low dose bath on the contralateral side of the tumor for CPPT compared to IMRT (see Figure 2a,b for patient 1 and Figure B1–B6 for patient 2–7). For the CPPT plans in Figure 2a,b, additionally, the proton contribution (Figure 2a1,b1) and the photon contribution (Figure 2a2,b2) to the total dose are visualized. For these treatments, the proton component of the combined plans typically reduces the low and medium dose bath to the healthy tissue, while photons are used to improve target coverage. The relative contribution of protons and photons to the total delivered target dose marginally depends on the patient geometry (Figure 2f). For the FHB CPPT, the proton contribution is in the range of 40%–64%. Furthermore, for all patients, the proton contribution for gantry CPPT is larger than with FHB CPPT (46%–84%). The spatial distribution of the proton and photon dose for the two CPPT approaches are visualized in Figure 3 for each patient with an example slice. Spatially, the weighting of the proton and photon component depends on the patient geometry, as well as on the approach chosen (FHB or gantry).

**FIGURE 4:**
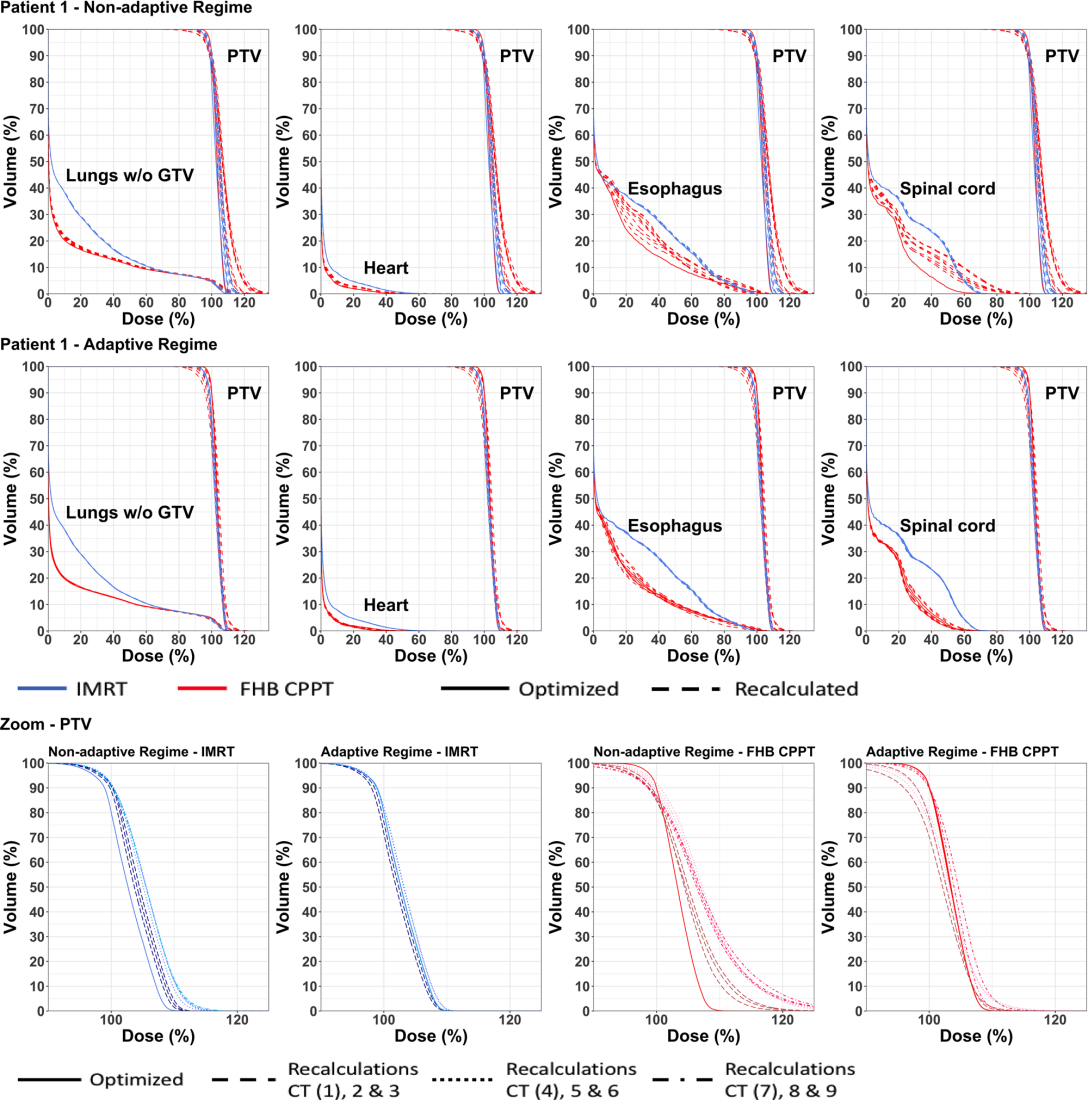
Dose–volume histograms (DVHs) for patient 1 in the non‐adaptive (top) and adaptive (middle) regime for the PTV together with healthy lungs, heart, spinal cord, and esophagus. The bottom panel shows a zoom of the PTV DVH, the line types and color tones are used for a separation between days

DVHs for the PTV and OARs are shown for the baseline case of patient 1 as solid lines in Figure 4, where there is reduction in the low and medium doses for all OARs, a trend also observed for all patients.

**FIGURE 5:**
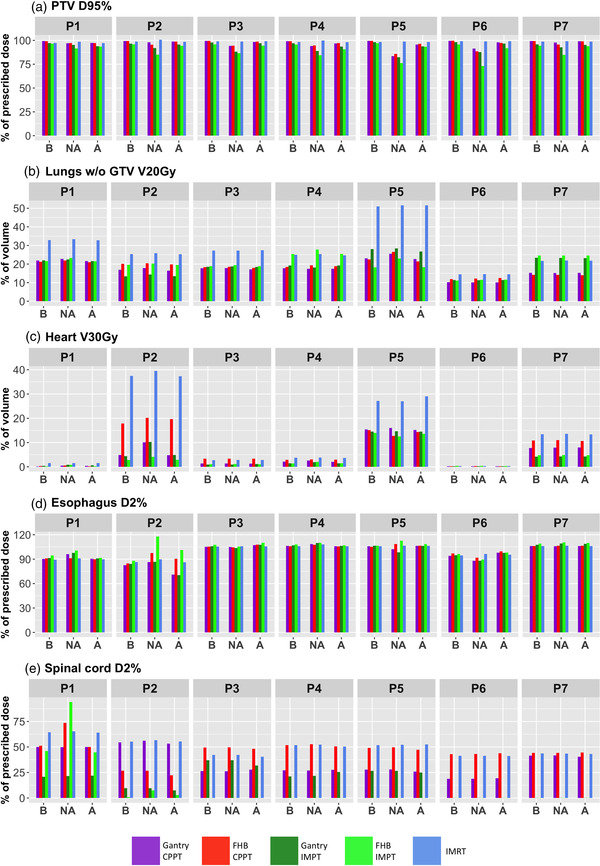
Dose parameters under investigation for the different patients in the baseline (B), non‐adaptive (NA), and adaptive (A) regime. For the adaptive and the non‐adaptive regimes, the values from CT0–CT9 and CT1–CT9, respectively, are averaged

Figure 5 tabulates selected dose parameters for all patients, while the mean over all patients can be found in Table B2. Additionally, differences between FHB CPPT and the other modalities are shown in Figure B7.

Results for the baseline scenario for all modalities can be summarized as follows:

1. FHB CPPT vs. IMRT: compared to the pure IMRT treatment, the CPPT treatment achieves a higher *D*_95%_ averaged over all patients (98.5%±0.6% vs. 99.4%±0.2%). The largest reductions in OAR dose can be seen in lung V_20Gy_ (28.2%±11.5% vs. 18.1%±3.8%) and V_30Gy_ to the heart (12.3%±14.6% vs. 7.2%±7.3%). *D*_2%_ to the esophagus (99.1%±8.7% vs. 99.4%±8.6%) and spinal cord (50.0%±8.3% vs. 45.1%±8.7%) are however quite comparable (Table B2) apart from for patient P1 and P2 (Figure 5e).
2. FHB CPPT vs. gantry CPPT: similar results have been observed for gantry CPPT as for FHB CPPT, apart from for the spinal cord where there is a substantial reduction in *D*_2%_ from 45.1%±8.7% to 35.2%±13.5% (Table B2) when using a gantry.
3. FHB CPPT vs. IMPT: compared to IMPT alone, FHB CPPT increases *D*_95%_ to the PTV (97.3%±0.8% vs. 99.4%±0.2%), while the heart V_30Gy_ can be reduced from 7.2%±7.3% to 3.7%±5.1% and *D*_2%_ of the spinal cord from 45.1%±8.7% to 16.7%±13.9% for IMPT alone (Table B2).

### 3.2 Non‐adaptive regime

The effect of anatomical changes on the baseline plans has been estimated by recalculating all plans on each repeat CT, thus simulating the effects of anatomical change. We define this as the *non‐adaptive* regime. In this regime, target coverage is especially affected for the CPPT and the IMPT treatment plans with the effect being largest when recalculation is performed on CT's acquired later in the treatment course. For patient 1, this can be seen in the DVH for the FHB CPPT treatment plan in the bottom panel of Figure 4. Figure 5 shows the reduced PTV *D*_95%_ for all patients of the CPPT and IMPT plans, with a larger effect on the IMPT plans. For the OARs, the results are comparable to the baseline regime, except for the spinal cord of patient 1 and esophagus of patient 2. Averaged over all patients, the PTV *D*_95%_ is substantially lowered by including all recalculated plans for the CPPT and the IMPT scenarios (Table B3). Results for the non‐adaptive regime can thus be summarized as follows:

1. FHB CPPT vs. IMRT: despite anatomical changes, IMRT plans achieve excellent target coverage (*D*_95%_ of 99.2%±0.8%) and robust sparing of OARs, while for FHB CPPT coverage is substantially reduced (93.1%±4.2%). For OARs, anatomical changes have less influence on FHB CPPT plans.
2. FHB CPPT vs. gantry CPPT: reduction on target coverage for gantry CPPT plans is similar (93.7%±5.1%) to that of FHB CPPT, although is slightly more robust than the FHB for OAR doses.
3. FHB CPPT vs. IMPT: anatomical changes have the largest impact on the IMPT alone plans, with PTV *D*_95%_ reducing to 83.1%±6.3% for FHB IMPT and 89.6%±4.2% for gantry IMPT. For the spinal cord and the esophagus, the FHB IMPT is for most patients more sensitive to anatomical changes than any CPPT scenario.

### 3.3 Adaptive regime

An adaptive treatment approach has been approximated by reoptimizing the plans on one repeat CT of each day when repeat CTs were acquired. However, in order to then simulate the effect of variations in breath‐holds during treatment, these reoptimized plans were then *recalculated* on the remaining two repeat CTs acquired on the same day. Results for this regime for patient 1 are shown in the middle panel of Figure 4 for recalculations of the FHB CPPT scenario. As can be seen, results are much closer to the baseline plan than for the non‐adaptive regime. Figure 5 shows results for all scenarios and patients, which can be summarized as follows:

1. FHB CPPT vs. IMRT: for the adaptive regime, PTV *D*_95%_ is comparable between FHB CPPT and IMRT (97.8% ± 1.0% vs. 98.5% ± 0.7%) and lung V_20Gy_ (17.9% ± 3.4% vs. 28.3% ± 11.7%) and heart V_30Gy_ (7.4% ± 7.6% vs. 12.6% ± 14.9%) are substantially reduced to levels similar to the baseline case (Table B4).
2. FHB CPPT vs. gantry CPPT: dose indices are comparable to the baseline regime, with only the spinal cord having a substantially lower value for gantry compared to FHB CPPT (Table B4).
3. FHB CPPT vs. IMPT: compared to the IMPT alone plans, CPPT results are close to those of the baseline scenario (Table B4).

### 3.4 Impact of range robust optimization

DVHs for range robust optimized (±3%, ±5%, and ±7%) IMRT and FHB CPPT scenarios for two example patients (P2 and P6) and for non‐adaptive and adaptive regimes are shown in Figure B8–B11. For the adaptive regime, range robust optimization is obsolete, as target coverage is always preserved also for plans recalculated on the repeat CTs of the same day. For the non‐adaptive regime however, range robust optimization helps to recover target coverage. However, for CPPT, the optimizer starts to substantially increase the photon component, compromising the benefit of CPPT. Generally, it can be seen that range robust optimization helps to recover the target coverage for the CPPT, but OAR doses approach the levels of the IMRT plan, especially when higher range uncertainties are assumed. As would be expected, range robust optimization for IMRT adds little to their already high robustness to anatomical changes.

### 3.5 NTCP

Calculated NTCPs for all plans optimized on the planning CT (baseline regime) are shown for all patients in Table 1. The probability for acute radiation pneumonitis can be reduced with FHB CPPT compared to IMRT by 1.3%–17.2% depending on the patient, with the largest reduction being observed for patients with the largest tumors (P1 and P5). NTCP reductions for gantry CPPT, as well as for IMPT alone treatments are comparable to reductions for the FHB CPPT. Moreover, the predicted 2‐year mortality is also reduced for the FHB CPPT compared to IMRT in the range of 0.6%–5.6%, although even higher reductions are observed for gantry CPPT or IMPT for which NTCP reductions can reach up to 13.0% or 22.6% respectively. Compared to IMRT, on average, reductions over all patients were 2.5%, 4.8%, 7.4%, 8.0% for FHB CPPT, gantry CPPT, gantry IMPT, and FHB IMPT respectively. For grade 2 esophageal toxicities, FHB CPPT reduces NTCP by between 1.6%–10.6% compared to IMRT, with higher reductions being observed once again for gantry CPPT and IMPT only plans. Finally, for both the non‐adaptive and adaptive regime, the NTCP values can be found in Tables B5 and B6. Due to the dependence on mean dose of all the NTCP models used here, little differences are found between the non‐adaptive and adaptive regimes.

**TABLE 1:** NTCPs for radiation pneumonitis, 2‐year mortality, and dysphagia of grade 2 or higher for the different treatment plans and patients, together with the reduction compared to the intensity‐modulated radiation therapy (IMRT) plan

NTCP [%]	Patient	IMRT	FHB CPPT	Red. to IMRT	Gantry CPPT	Red. to IMRT	Gantry IMPT	Red. to IMRT	FHB IMPT	Red. to IMRT
Radiation pneumonitis	P1	13.7	7.7	−6.0	8.3	−5.4	7.2	−6.4	7.3	−6.3
	P2	10.1	6.2	−3.9	6.5	−3.5	4.1	−6.0	5.7	−4.4
	P3	8.7	5.2	−3.5	4.9	−3.8	4.4	−4.3	4.9	−3.9
	P4	8.7	6.3	−2.3	5.9	−2.7	5.3	−3.3	7.4	−1.3
	P5	26.9	9.7	−17.2	9.6	−17.3	9.2	−17.7	6.2	−20.7
	P6	4.9	3.6	−1.3	3.2	−1.7	2.9	−2.0	3.1	−1.8
	P7	7.3	4.8	−2.5	4.7	−2.6	5.3	−2.0	5.8	−1.4
	**Avg**	**11.5**	**6.2**	−**5.2**	**6.2**	−**5.3**	**5.5**	−**6.0**	**5.8**	−**5.7**
2‐year mortality	P1	47.5	44.8	−2.7	43.6	−3.9	43.6	−4.0	42.5	−5.0
	P2	58.4	54.3	−4.1	45.4	−13.0	38.3	−20.1	35.8	−22.6
	P3	35.4	34.9	−0.6	32.9	−2.6	31.5	−4.0	30.7	−4.8
	P4	60.4	59.1	−1.3	58.5	−1.9	56.1	−4.3	56.6	−3.8
	P5	78.5	72.9	−5.6	72.4	−6.1	71.7	−6.8	71.2	−7.3
	P6	31.4	30.6	−0.9	29.4	−2.0	29.0	−2.4	29.4	−2.0
	P7	47.1	45.1	−2.0	43.3	−3.9	36.5	−10.6	36.9	−10.3
	**Avg**	**51.3**	**48.8**	−**2.5**	**46.5**	−**4.8**	**43.8**	−**7.4**	**43.3**	−**8.0**
Esophageal toxicity	P1	31.9	21.3	−10.6	19.9	−12.1	12.8	−19.1	21.4	−10.5
	P2	28.9	18.3	−10.6	13.6	−15.3	7.1	−21.8	13.2	−15.7
	P3	46.2	41.9	−4.4	39.0	−7.2	33.7	−12.5	29.6	−16.6
	P4	9.0	6.6	−2.4	5.5	−3.5	4.3	−4.8	5.8	−3.2
	P5	53.9	45.8	−8.1	43.0	−10.9	37.3	−16.7	40.1	−13.9
	P6	26.1	17.3	−8.8	11.3	−14.8	9.6	−16.5	12.6	−13.5
	P7	52.6	51.0	−1.6	51.2	−1.4	45.6	−7.0	48.2	−4.4
	**Avg**	**35.5**	**28.9**	−**6.6**	**26.2**	−**9.3**	**21.5**	−**14.0**	**24.4**	−**11.1**

## 4 DISCUSSION

The management of NSCLC is challenging for radiotherapy in general and proton therapy has been suggested to improve radiotherapy treatments for NSCLC patients. The clinical benefit of protons is currently been evaluated in a phase III trial (ClinicalTrials.gov: NCT0199381). However, due to the higher costs, proton therapy is still not available to all patients whom could benefit. In this study, we have conducted a first investigation of the potential of combining proton–photon treatments, through simultaneous optimization, for lung cancer patients. In particular, we investigated how such combined treatments compare to single modality treatments, not just for the baseline case, but also in the presence of inter‐fractional anatomic changes and breath‐hold variability.

In the simultaneously optimized CPPT treatments calculated here, the proton component has been found to mainly spare healthy tissue (reducing the dose bath), while photons help to improve plan conformity. Around half of the dose is delivered by protons for the FHB approach (Figure 2f). If the proton component could be delivered on a gantry though, the proton contribution was found to increase for all patients, as the additional flexibility in beam angles leads to a better dose conformity due to proton alone, and thus the optimizer tends towards a more predominant proton solution.

However, CPPT treatments with a FHB still show substantial improvements to IMRT‐only treatments for NSCLC patients, especially in reducing the low and medium doses to OARs and the healthy tissue. In contrast, for the high doses to OARs, the plan quality of CPPT heavily depends on the patient geometry. For the spinal cord for example, the CPPT plan delivers higher doses than IMRT in 2/7 cases. These results are in line with the previous conclusions for HN cancer patients, for which the doses to OARs could also be reduced compared to IMRT.^12^

The benefit of using a gantry instead of a FHB for CPPT can help to further reduce the dose to the spinal cord for six out of seven patients in our dataset, depending on the relative location of the spinal cord to the tumor. In general, however, the benefit of using a gantry as part of CPPT is questionable with IMPT only plans mainly improving the dose delivered to the spinal cord and the heart, whereas target coverage is better for CPPT.

From the repeat CT datasets used here, it could be observed that inter‐fractional anatomical changes were substantial for most patients. In particular, target coverage of both CPPT and IMPT is degraded if the original plan is recalculated on this changed anatomy. On the other hand, for the OARs, the influence is rather small. Unsurprisingly, IMRT treatment plans have been found to be robust to the same anatomic variations, while CPPT is more robust than IMPT only treatments. Nevertheless, our results show that adaption would be an advantage for most patients even when treated using a CPPT regime. In contrast, our results from combining CPPT with range robust optimization are inconclusive and require further work. Although it appears to make CPPT plans more robust to anatomical changes, at least for larger magnitudes of range uncertainty and the combined optimization used in this work, this is achieved by simply increasing the IMRT component of the treatment, thus effectively mitigating the advantages of the CPPT approach. It should be noted however, that the anatomical changes studied here are limited to three time‐points, and further investigations on data with more time‐points will be needed to check the time intervals for which adaption might be necessary. Additionally, as DIBH might not be feasible for all patients, the feasibility of CPPT in combination with motion mitigation approaches needs to be further investigated, together with the clinical feasibility of enhanced DIBH approaches.^38^

CPPT also shows promising results in terms of NTCP, with CPPT plans consistently reducing the probability for radiation pneumonitis compared to IMRT. This reduction is especially pronounced in the case of large tumors and IMPT only or gantry‐based CPPT do not bring additional benefit for this complication, indicating the potential effectiveness of a fixed beam concept. However, FHB CPPT is not so effective at reducing the probability of > = grade 2 esophageal toxicities in comparison to gantry CPPT or IMPT only plans. Important to mention, there is the possibility of even better gantry angles, as the comparison between FHB and gantry was not the main comparison of this work and to stay consistent, we chose the previously published beam configurations,^30^ which were considered in order to help plan robustness, provide clinically adequate dose homogeneity across the PTV and to spare the contralateral lung completely. The difficulty of finding the optimal beam angles using the example of Patient 4 are discussed in Supplement C. For the most severe NTCP studied (2‐year mortality), NTCP reductions for FHB CPPT compared to IMRT are also smaller in magnitude. However, for such a severe endpoint, even a small reduction could be seen as highly beneficial. The influences of inter‐fractional changes and breath‐hold variability on all the studied NTCP values are small, as all models are dependent on mean dose which is only marginally affected by anatomical changes.

Although the normal tissue sparing advantages of FHB CPPT in comparison to IMRT for NSCLC have been found to be somewhat less than those of gantry‐based IMPT‐only treatments, this approach could nevertheless be of interest due the simplicity of the machine and the very much reduced space that would be required for such a treatment room. Indeed, an FHB could also be potentially combined in an existing Linac vault. In addition, in this work, we have assumed no inclination angle for the fixed beam line. However, additional research is also needed in the future to determine whether there are possible additional benefits of inclined proton beam lines or other beam line configurations. An inclined beam line could give the chance to different beam angles for the fixed beam line approach. On the other hand, the optimal inclination angle is expected to vary for different indications and needs therefore more extensive research to find the optimum for multiple indications, which is beyond the scope of this paper. Additionally, an inclined beam line might increase the cost of the treatment compared to a horizontal beam line. Another option which could increase the flexibility of a fixed horizontal beam line substantially is an upright positioning of the patient, which lately gained interest again in the community and by vendors.^39^, ^40^, ^41^, ^42^

## 5 CONCLUSIONS

Optimally CPPT has the potential to increase the accessibility of NSCLC patients to the benefits of proton therapy. Such combined treatment plans improve the delivered dose compared to IMRT, with the plan quality being reduced only for certain OARs and for some patients in comparison to IMPT‐only plans. Furthermore, NTCP analysis has indicated that the probabilities for radiation pneumonitis, > = grade 2 esophageal toxicity and for 2‐year mortality can be reduced for CPPT compared to IMRT, and that CPPT can reduce the sensitivity of the plan to anatomical changes. Nevertheless, in the presence of large inter‐fractional changes, adaptive therapy regimes may be necessary in combination with CPPT to preserve target coverage.

## CONFLICT OF INTEREST

The authors declare that there is no conflict of interest that could be perceived as prejudicing the impartiality of the research reported.

## Supporting information

Table A1: Field angles used for the gantry CPPT and the gantry IMPT treatment plans 30.
Figure B1: Treatment plans for patient 2 (PTV in red).
Figure B2: Treatment plans for patient 3 (PTV in red).
Figure B3: Treatment plans for patient 4 (PTV in red).
Figure B4: Treatment plans for patient 5 (PTV in red).
Figure B5: Treatment plans for patient 6 (PTV in red).
Figure B6: Treatment plans for patient 7 (PTV in red).
Table B1: DVH parameters in the adaptive regime for P1–P7 and the different modalities averaged over all nine CTs (CT1–CT9).
Table B2: Averaged dose parameters over all patients in the ideal regime.
Table B3: Averaged dose parameters over all patients and CT0–CT9 in the non‐adaptive regime.
Table B4: Averaged dose parameters over all patients and CT1–CT9 in the adaptive regime.
Figure B7: Difference of FHB CPPT to other modalities for the baseline (B), non‐adaptive (NA), and the adaptive (A) regime
Figure B8: Patient 2 DVHs for IMRT and FHB CPPT scenarios without and with (±3%, ±5%, and ±7%) range robust optimization in the non‐adaptive regime.
Figure B9: Patient 2 DVHs for IMRT and FHB CPPT scenarios without and with (±3%, ±5%, and ±7%) range robust optimization in the adaptive regime.
Figure B10: Patient 6 DVHs for IMRT and FHB CPPT scenarios without and with (±3%, ±5%, and ±7%) range robust optimization in the non‐adaptive regime
Figure B11: Patient 6 DVHs for IMRT and FHB CPPT scenarios without and with (±3%, ±5%, and ±7%) range robust optimization in the adaptive regime.
Table B5: NTCP values for the non‐adaptive regime the values for each patient are the average over CT0–CT9.
Table B6: NTCP values for the adaptive regime the values for each patient are the average over CT1–CT9
Figure C1: (A) and (B) IMPT gantry dose distributions for Patient 4 using two different beam configurations
