# Supplementary material for: Combined proton–photon therapy for non‐small cell lung cancer
Source: Med Phys. 2022 May 25;49(8):5374–86. doi: 10.1002/mp.15715 (PMC9544482; doi:10.1002/mp.15715)
Supplement: Supplementary file 1 — Table A1: Field angles used for the gantry CPPT and the gantry IMPT treatment plans 30. Figure B1: Treatment plans for patient 2 (PTV in red). Figure B2: Treatment plans for patient 3 (PTV in red). Figure B3: Treatment plans for patient 4 (PTV in red). Figure B4: Treatment plans for patient 5 (PTV in red). Figure B5: Treatment plans for patient 6 (PTV in red). Figure B6: Treatment plans for patient 7 (PTV in red). Table B1: DVH parameters in the adaptive regime for P1–P7 and the different modalities averaged over all nine CTs (CT1–CT9). Table B2: Averaged dose parameters over all patients in the ideal regime. Table B3: Averaged dose parameters over all patients and CT0–CT9 in the non‐adaptive regime. Table B4: Averaged dose parameters over all patients and CT1–CT9 in the adaptive regime. Figure B7: Difference of FHB CPPT to other modalities for the baseline (B), non‐adaptive (NA), and the adaptive (A) regime Figure B8: Patient 2 DVHs for IMRT and FHB CPPT scenarios without and with (±3%, ±5%, and ±7%) range robust optimization in the non‐adaptive regime. Figure B9: Patient 2 DVHs for IMRT and FHB CPPT scenarios without and with (±3%, ±5%, and ±7%) range robust optimization in the adaptive regime. Figure B10: Patient 6 DVHs for IMRT and FHB CPPT scenarios without and with (±3%, ±5%, and ±7%) range robust optimization in the non‐adaptive regime Figure B11: Patient 6 DVHs for IMRT and FHB CPPT scenarios without and with (±3%, ±5%, and ±7%) range robust optimization in the adaptive regime. Table B5: NTCP values for the non‐adaptive regime the values for each patient are the average over CT0–CT9. Table B6: NTCP values for the adaptive regime the values for each patient are the average over CT1–CT9 Figure C1: (A) and (B) IMPT gantry dose distributions for Patient 4 using two different beam configurations [file MP-49-5374-s001.pdf]

Supplementary material

for the publication

"Combined proton-photon therapy for non-small cell lung cancer"

Supplement A

For CPPT treatment planning, the cumulative dose of IMRT and IMPT plans are simultaneously optimized by solving the following optimization problem:

$$\underset{{}^{\gamma}\mathbf{x},\,{}^{p}\mathbf{x}}{\text{minimize}} \quad \sum_k p_k\, f(\,{}^{\gamma}\mathbf{d}^k + {}^{p}\mathbf{d}^k\,) \tag{A.1}$$

$$\text{subject to} \quad {}^{\gamma}d_i^k = \sum_j {}^{\gamma}D_{ij}^k\, {}^{\gamma}x_j \qquad \forall i, k \tag{A.2}$$

$${}^{p}d_i^k = \sum_l {}^{p}D_{il}^k\, {}^{p}x_l \qquad \forall i, k \tag{A.3}$$

$${}^{\gamma}x_j\,,\ {}^{p}x_l \ \geq\ 0 \qquad \forall j, l \tag{A.4}$$

for which the objective function f defines the clinical goals and is evaluated for the cumulative dose of photons and protons for all defined error scenarios k. The dose delivered by photons and protons in an error scenario k is given by ${}^{\gamma}\mathbf{d}^k$ and ${}^{p}\mathbf{d}^k$. For photons ${}^{\gamma}D_{ij}^k$, respectively for protons ${}^{p}D_{il}^k$, represent the elements of the dose-influence matrices and denote the dose contribution of a beamlet j and a pencil beam l to a voxel i for unit intensities in an error scenario k. These dose-influence matrices are calculated with the open-source radiation treatment planning toolkit matRad [32]. Moreover, the intensities of a beamlet j and a pencil beam l are designated by ${}^{\gamma}x_j$ and ${}^{p}x_l$. The proton dose ${}^{p}\mathbf{d}^k$ includes a constant relative biological effectiveness (RBE) factor of 1.1. The full objective function f(d) is defined as:

$$f(\mathbf{d}) = \frac{1}{N_{PTV}} \sum_{i \in PTV} [8(70 - d_i)_+^2 + 5(d_i - 74.9)_+^2] \tag{A.5}$$

$$+ \frac{1}{N_{SC}} \sum_{i \in SC} (d_i - 45)_+^2 \tag{A.6}$$

$$+ \frac{1}{N_E} \sum_{i \in E} (d_i - 74)_+^2 \tag{A.7}$$

$$+ \frac{1}{N_L} \sum_{i \in L} d_i \tag{A.8}$$

$$+ \frac{1}{N_{HT}} \sum_{i \in HT} (d_i - 35)_+^2 \tag{A.9}$$

$$+ \frac{1}{N_{HT}} \sum_{i \in HT} d_i \tag{A.10}$$

with d_i representing the dose at voxel i and N_{VOI} denotes the number of voxels in the respective volume of interest (VOI). In the present objective function, the VOIs are the PTV, the spinal cord (SC), the esophagus (E), the lung (L) and the healthy tissue (HT). An additional conformity objective is defined within 1 cm margin (Ma) around the PTV:

$$d_i^{max} \leq\ 70 - z_i\ \ (70 - 35) \qquad \forall i \in Ma \tag{A.11}$$

using a maximum dose d_i^{max} and z_i the Euclidean distance in cm of a HT voxel from the PTV contour.

In case of the non-robust optimization only one scenario is optimized, having k=1 and p_k=1. To achieve robust plans against different uncertainties, a hybrid robust planning approach is applied [33], for which, a PTV margin is added to the CTV to account for setup uncertainties. The range uncertainties are mitigated with stochastic optimization by evaluating the cumulative dose for all error scenarios k and minimizes the sum of the objective function values weighted by an importance factor p_k. In this work, three scenarios were used, representing the nominal scenario (p_k=0.5) and an under- and overshoot scenario (p_k=0.25) [34].

The dose influence matrices for the photons and protons, calculated in matRad, have a beamlet size of 5 x 5 mm^2 for the 6 MV photon beamlets and a lateral/longitudinal spot spacing of 5 mm/3 mm for the proton pencil beams. A generic proton machine using energies from 31.7-236.1 MeV is assumed to deliver a gaussian lateral beam profile with an initial lateral spread (σ) at patient surface of 2.3 mm for the highest energy and 5 mm for the lowest energy. The photon beamlet intensities and proton pencil beam intensities are simultaneously optimized based on their cumulative dose with an in-house implementation of the L-BFGS quasi-Newton method.

	**Field 1 [°]**	**Field 2 [°]**	**Field 3 [°]**
**Patient 1**	10	200	225
**Patient 2**	150	170	190
**Patient 3**	200	240	340
**Patient 4**	200	240	340
**Patient 5**	200	245	355
**Patient 6**	20	60	110
**Patient 7**	220	240	260

Table A1: Field angles used for the gantry CPPT and the gantry IMPT treatment plans [30].

Supplement B

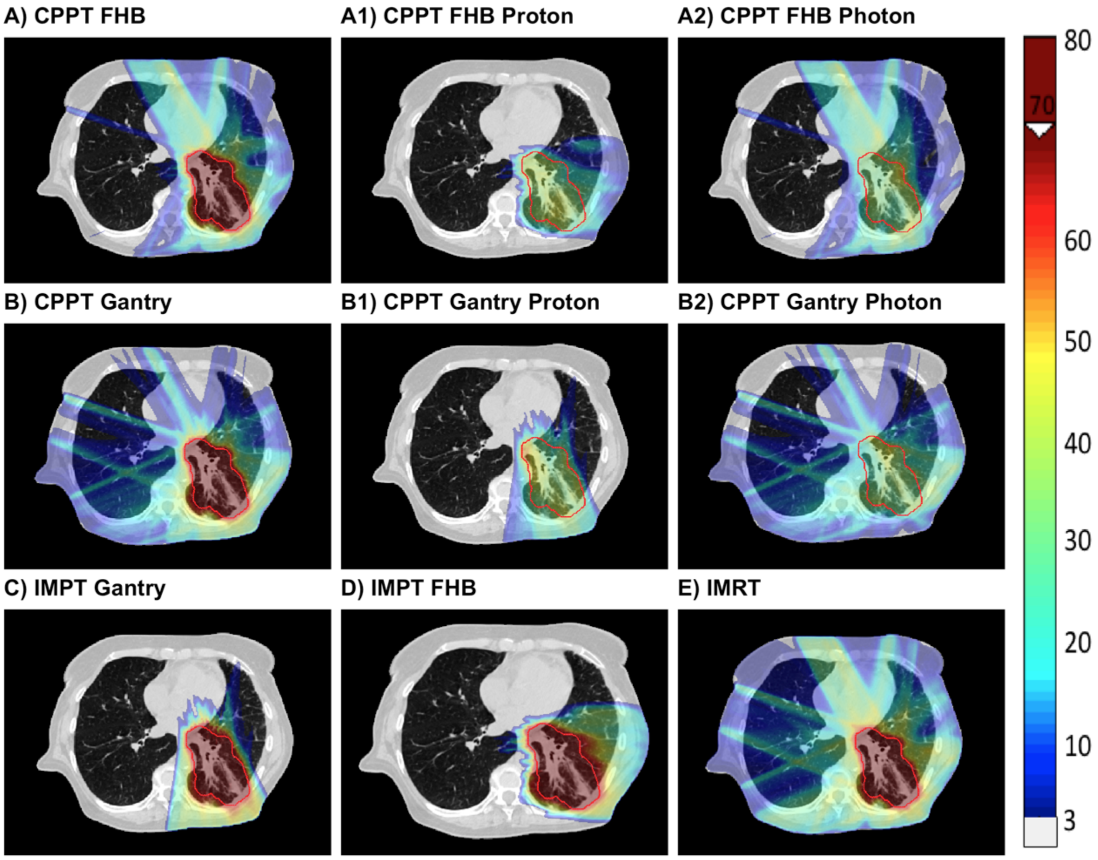

Figure B1: Treatment plans for patient 2 (PTV in red).

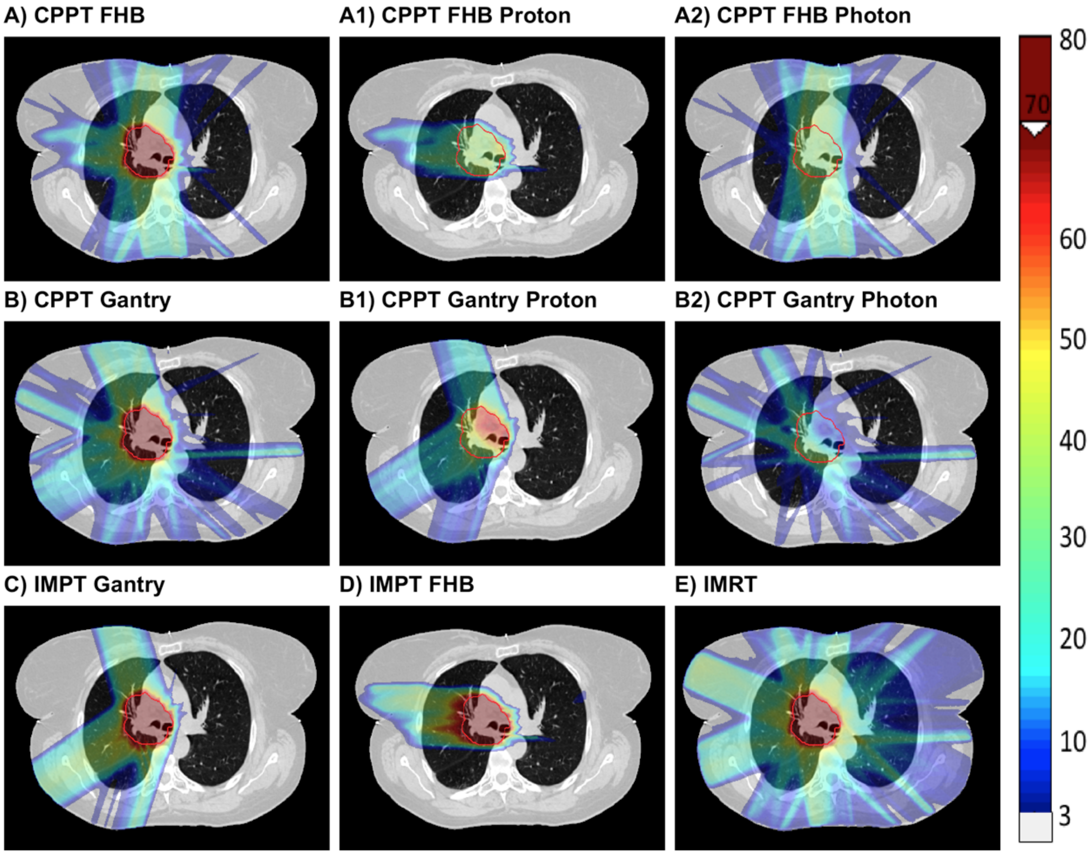

Figure B2: Treatment plans for patient 3 (PTV in red).

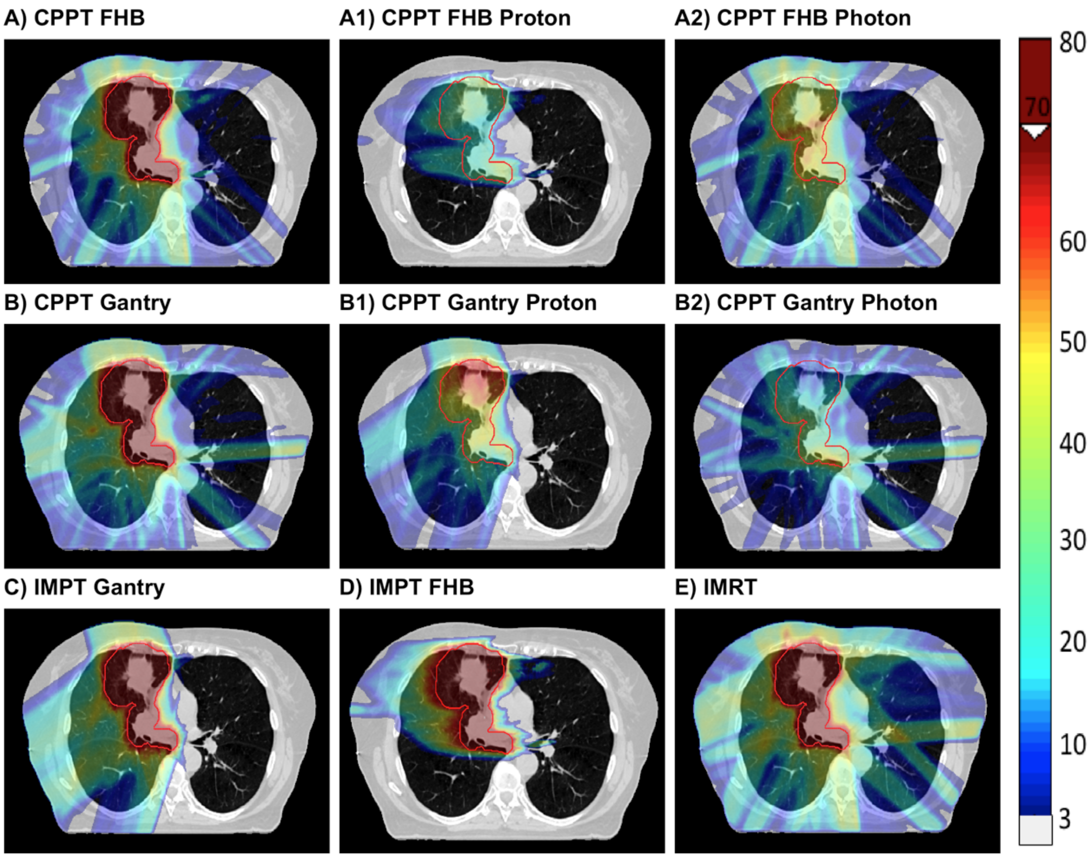

Figure B3: Treatment plans for patient 4 (PTV in red).

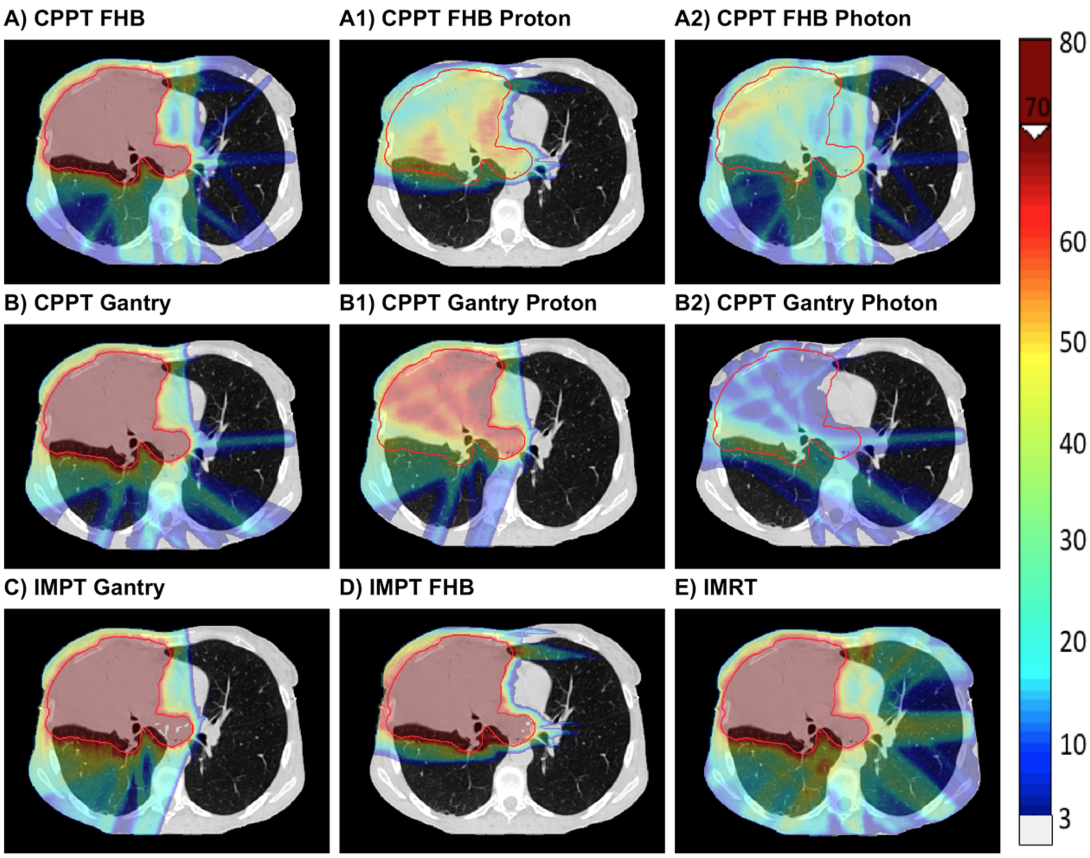

Figure B4: Treatment plans for patient 5 (PTV in red).

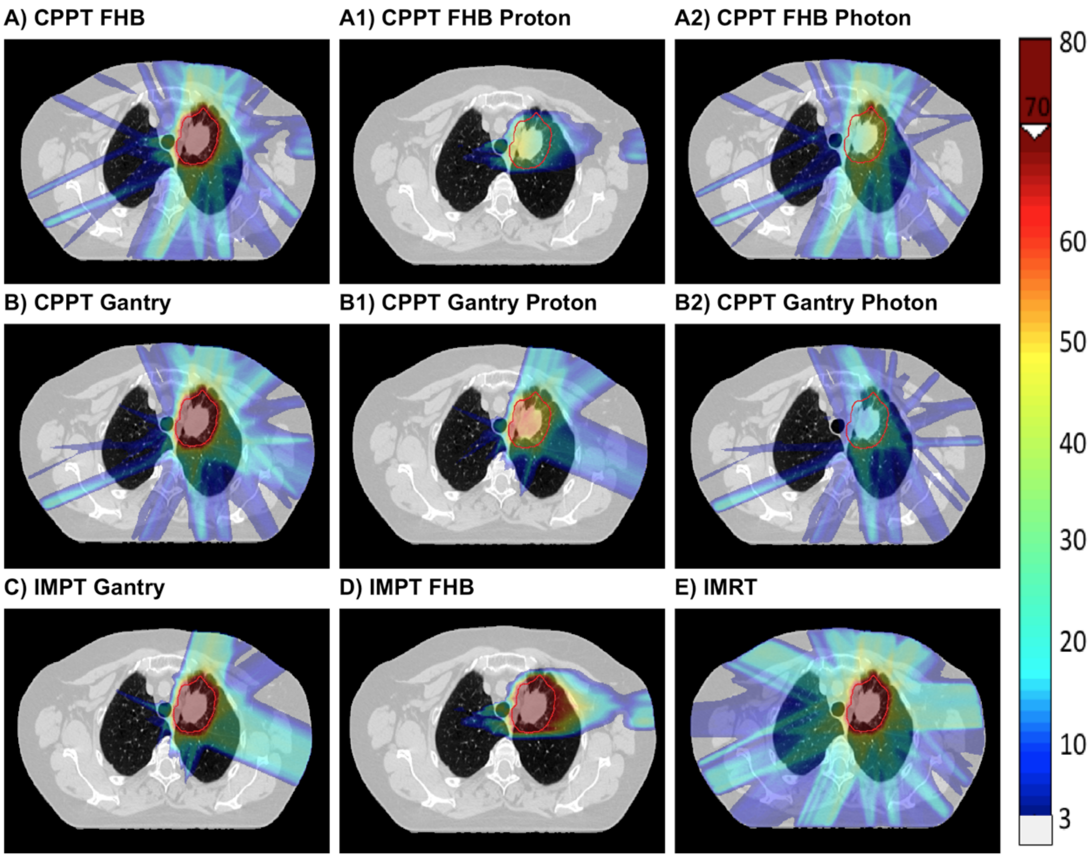

Figure B5: Treatment plans for patient 6 (PTV in red).

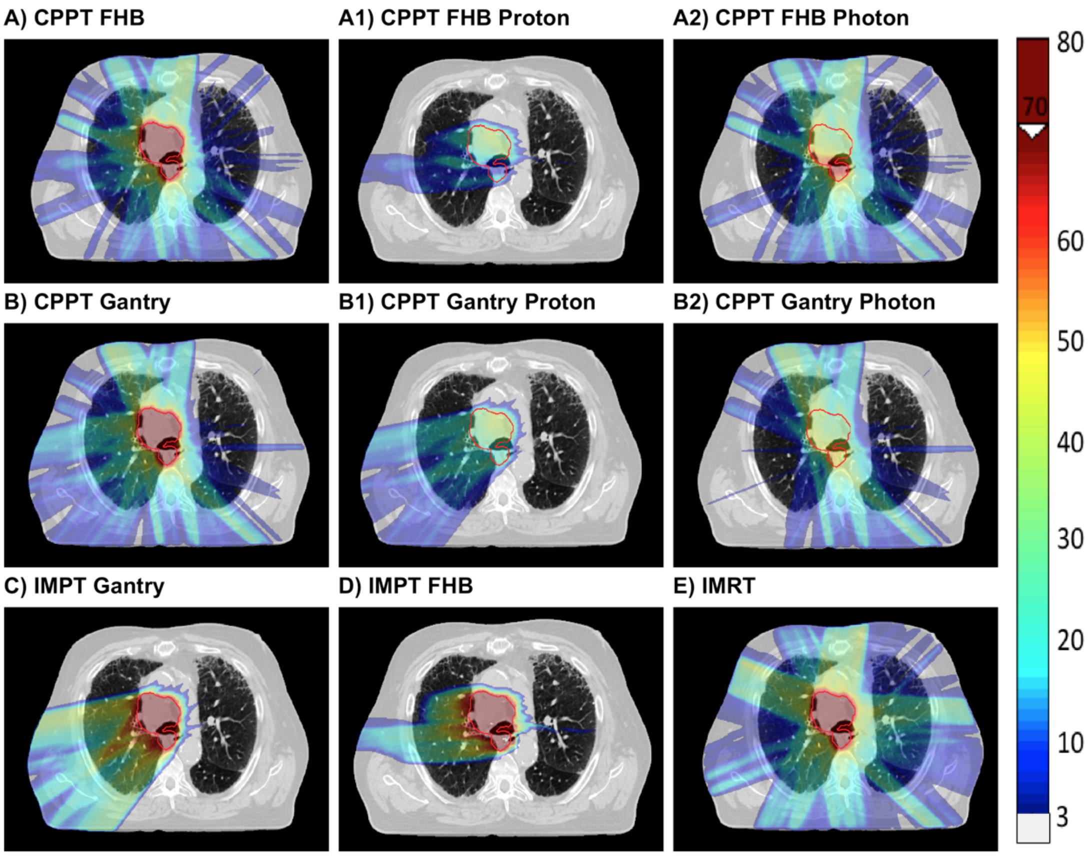

Figure B6: Treatment plans for patient 7 (PTV in red).

PTV:									
**D95%**	**P1**	**P2**	**P3**	**P4**	**P5**	**P6**	**P7**	**Avg**	**Std**
**IMRT**	97.3	98.6	99.1	98.2	98.3	99.2	98.8	98.5	0.7
**CPPT (FHB)**	97.3	98.7	98.7	97.1	96.4	97.5	99.0	97.8	1.0
**IMPT (FHB)**	93.5	94.3	94.5	90.7	93.4	91.7	93.9	93.1	1.4
**CPPT (Gantry)**	97.3	98.7	98.4	96.7	95.7	98.0	99.1	97.7	1.2
**IMPT (Gantry)**	93.9	95.6	97.0	93.5	93.7	96.6	95.3	95.1	1.4
**Lungs w/o GTV:**									
**V20Gy**	**P1**	**P2**	**P3**	**P4**	**P5**	**P6**	**P7**	**Avg**	**Std**
**IMRT**	32.8	25.2	27.4	24.7	51.5	14.5	21.8	28.3	11.7
**CPPT (FHB)**	21.0	19.8	17.9	18.8	21.4	12.5	14.0	17.9	3.4
**IMPT (FHB)**	21.5	19.5	18.9	25.4	18.3	11.6	24.6	20.0	4.6
**CPPT (Gantry)**	21.6	16.4	17.1	17.5	22.7	10.1	15.3	17.3	4.2
**IMPT (Gantry)**	21.6	13.5	18.4	19.3	26.7	11.4	23.2	19.2	5.4
**Heart:**									
**V30Gy**	**P1**	**P2**	**P3**	**P4**	**P5**	**P6**	**P7**	**Avg**	**Std**
**IMRT**	1.6	37.3	2.9	3.7	29.1	0.3	13.4	12.6	14.9
**CPPT (FHB)**	0.2	19.6	3.4	3.0	14.3	0.3	10.6	7.4	7.6
**IMPT (FHB)**	0.2	2.9	1.1	1.5	13.6	0.4	4.8	3.5	4.7
**CPPT (Gantry)**	0.4	4.8	1.3	2.1	15.2	0.3	8.0	4.6	5.4
**IMPT (Gantry)**	0.6	4.9	1.1	1.5	14.5	0.3	4.3	3.9	5.0
**Spinal cord:**									
**D2%**	**P1**	**P2**	**P3**	**P4**	**P5**	**P6**	**P7**	**Avg**	**Std**
**IMRT**	64.2	55.5	40.4	50.4	52.5	41.3	43.1	49.6	8.7
**CPPT (FHB)**	50.1	22.2	48.1	50.6	47.3	43.8	44.6	43.8	9.9
**IMPT (FHB)**	44.7	3.0	0.1	0.1	0.1	0.1	0.1	6.9	16.7
**CPPT (Gantry)**	50.0	53.2	27.8	27.7	25.8	19.4	40.4	34.9	13.1
**IMPT (Gantry)**	21.9	7.4	31.7	25.5	24.9	0.1	0.1	15.9	13.1
**Esophagus:**									
**D2%**	**P1**	**P2**	**P3**	**P4**	**P5**	**P6**	**P7**	**Avg**	**Std**
**IMRT**	89.8	86.3	105.7	105.8	106.3	95.4	106.1	99.3	8.7
**CPPT (FHB)**	89.7	90.6	108.0	105.7	106.5	99.6	106.5	101.0	7.8
**IMPT (FHB)**	91.7	101.1	110.2	107.0	108.6	98.2	109.9	103.8	7.0
**CPPT (Gantry)**	90.5	71.1	107.2	106.0	106.4	97.9	106.1	97.9	13.3
**IMPT (Gantry)**	91.0	70.4	107.7	106.4	106.3	97.7	108.9	98.3	13.9

Table B1: DVH parameters in the adaptive regime for P1-P7 and the different modalities averaged over all nine CTs (CT1-CT9). Values in percentage of prescribed dose. The last two columns are the average and standard deviation over the patients.

Baseline regime	Structure	Parameter	IMRT	FHB CPPT	Gantry CPPT	FHB IMPT	Gantry IMPT
	PTV	D95%	98.5 ± 0.6 %	99.4 ± 0.2 %	99.4 ± 0.2%	95.9 ± 0.8 %	97.3 ± 0.8 %
	Lungs w/o GTV	V20Gy	28.2 ± 11.5 %	18.1 ± 3.8 %	17.5 ± 4.3 %	19.9 ± 4.7 %	19.4 ± 5.7 %
	Heart	V30Gy	12.3 ± 14.6 %	7.2 ± 7.3 %	4.5 ± 5.5 %	3.5 ± 4.9 %	3.7 ± 5.1 %
	Esophagus	D2%	99.1 ± 8.7 %	99.4 ± 8.6 %	98.6 ± 9.7 %	101.5 ± 8.4 %	99.6 ± 9.5 %
	Spinal cord	D2%	50.0 ± 8.3%	45.1 ± 8.7 %	35.2 ± 13.5 %	6.8 ± 17.3 %	16.5 ± 13.8 %

Table B2: Averaged dose parameters over all patients in the ideal regime.

Non-adaptive regime	Structure	Parameter	IMRT	FHB CPPT	Gantry CPPT	FHB IMPT	Gantry IMPT
	PTV	D95%	99.2 ± 0.8 %	93.1 ± 4.2 %	93.7 ± 5.1%	83.1 ± 6.3 %	89.6 ± 4.2 %
	Lungs w/o GTV	V20Gy	28.6 ± 11.6 %	19.0 ± 4.8 %	18.1 ± 5.0 %	21.4 ± 5.2 %	19.6 ± 5.7 %
	Heart	V30Gy	12.6 ± 15.1 %	7.3 ± 7.5 %	5.5 ± 6.0 %	3.7 ± 4.2 %	4.7 ± 5.6 %
	Esophagus	D2%	100.6 ± 7.9 %	101.1 ± 7.6 %	98.9 ± 8.9 %	106.7 ± 9.3 %	99.1 ± 9.3 %
	Spinal cord	D2%	50.5 ± 8.8%	48.5 ± 13.9 %	35.4 ± 13.9 %	14.6 ± 35.1 %	16.7 ± 13.9 %

Table B3: Averaged dose parameters over all patients and CT0-CT9 in the non-adaptive regime.

Adaptive regime	Structure	Parameter	IMRT	FHB CPPT	Gantry CPPT	FHB IMPT	Gantry IMPT
	PTV	D95%	98.5 ± 0.6 %	97.8 ± 1.0 %	97.7 ± 1.2%	93.1 ± 1.4 %	95.1 ± 1.4 %
	Lungs w/o GTV	V20Gy	28.3 ± 11.7 %	17.9 ± 3.4 %	17.3 ± 4.2 %	20.0 ± 4.6 %	19.2 ± 5.4 %
	Heart	V30Gy	12.6 ± 14.9 %	7.4 ± 7.6 %	4.6 ± 5.4 %	3.5 ± 4.7 %	3.9 ± 5.0 %
	Esophagus	D2%	99.3 ± 8.7 %	100.9 ± 7.8 %	97.9 ± 13.3 %	103.8 ± 7.0 %	98.3 ± 13.9 %
	Spinal cord	D2%	49.6 ± 8.7%	43.8 ± 9.9 %	34.9 ± 13.1 %	6.9 ± 16.7 %	15.9 ± 13.1 %

Table B4: Averaged dose parameters over all patients and CT1-CT9 in the adaptive regime.

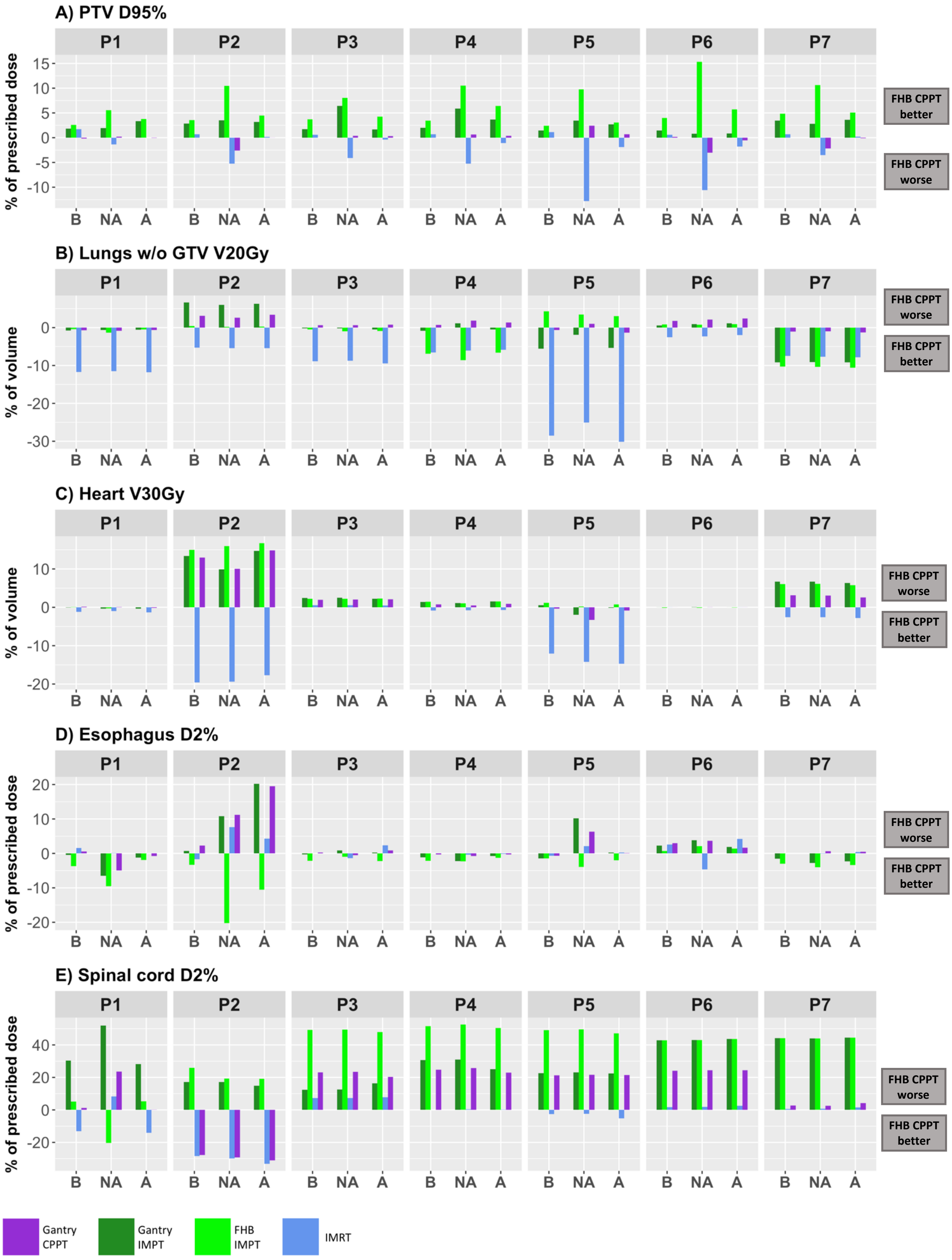

Figure B7: Difference of FHB CPPT to other modalities for the baseline (B), non-adaptive (NA), and the adaptive (A) regime

Patient 2 – Non-adaptive regime

No range robust optimization:

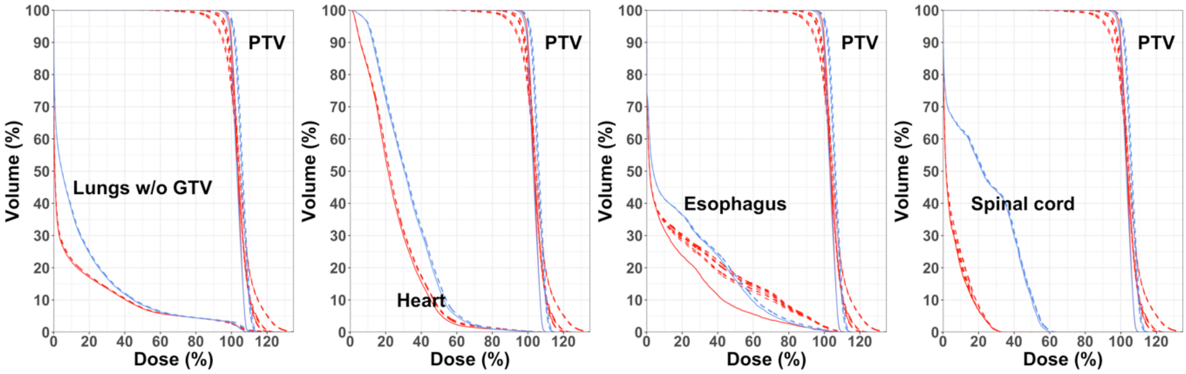

3% over- and undershoot:

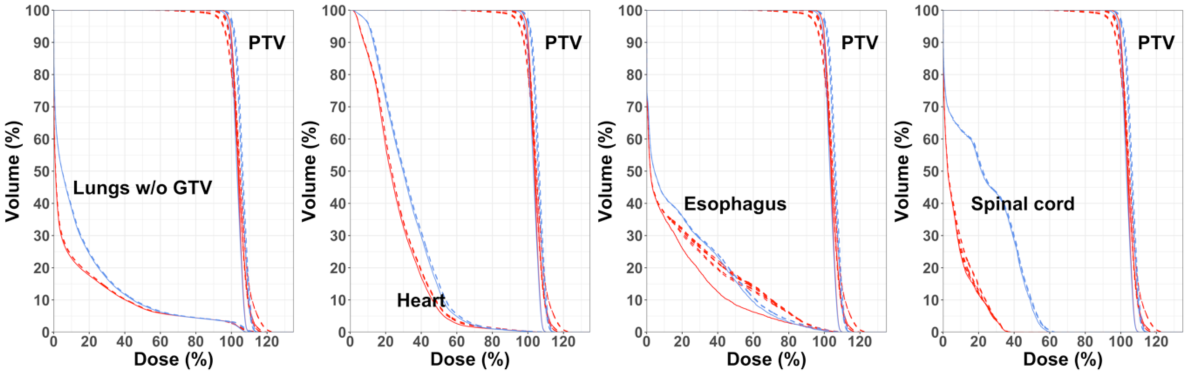

5% over- and undershoot:

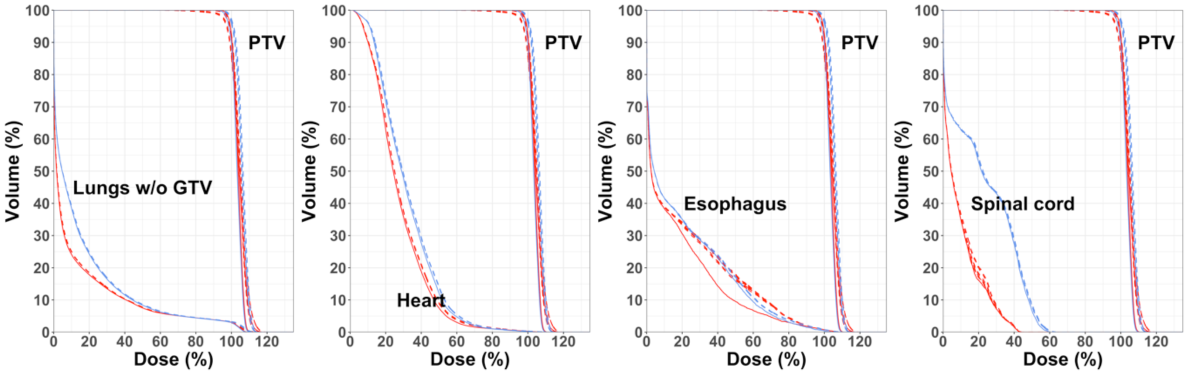

7% over- and undershoot

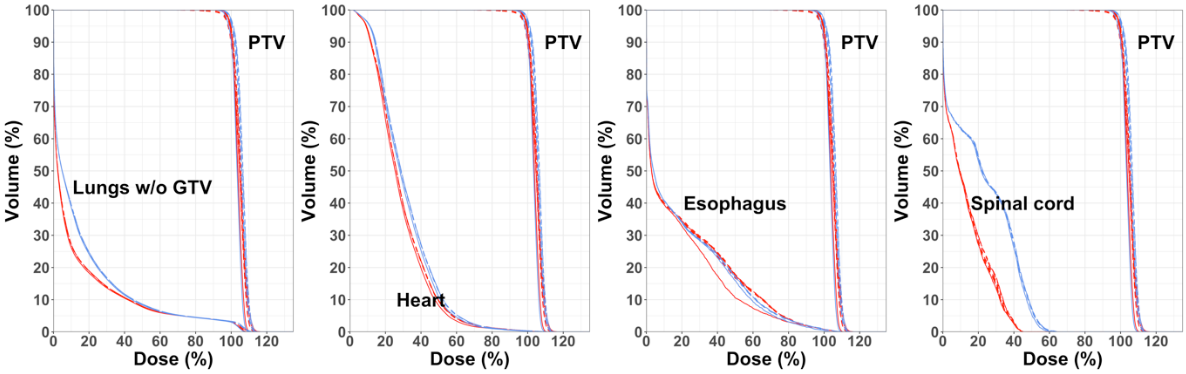

Figure B8: Patient 2 DVHs for IMRT and FHB CPPT scenarios without and with (±3%, ±5% and ±7%) *range robust optimization in the non-adaptive regime.*

Patient 2 – Adaptive regime

No range robust optimization:

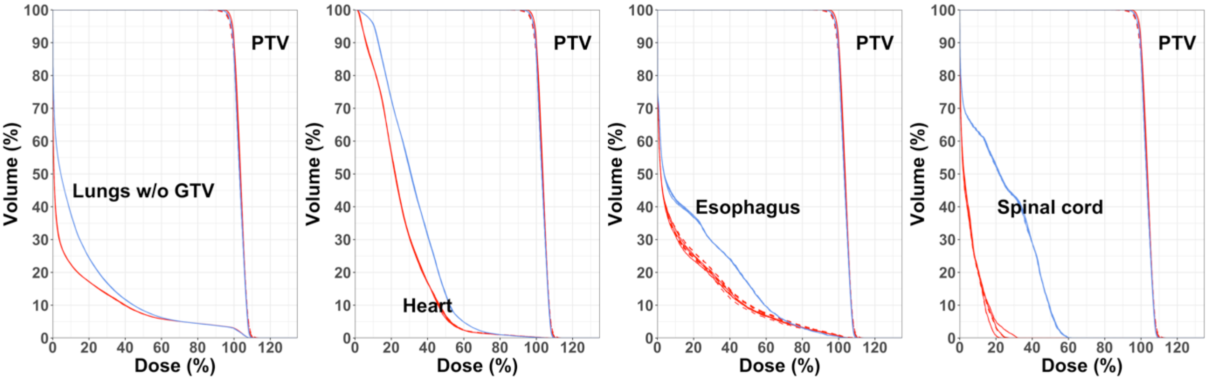

3% over- and undershoot:

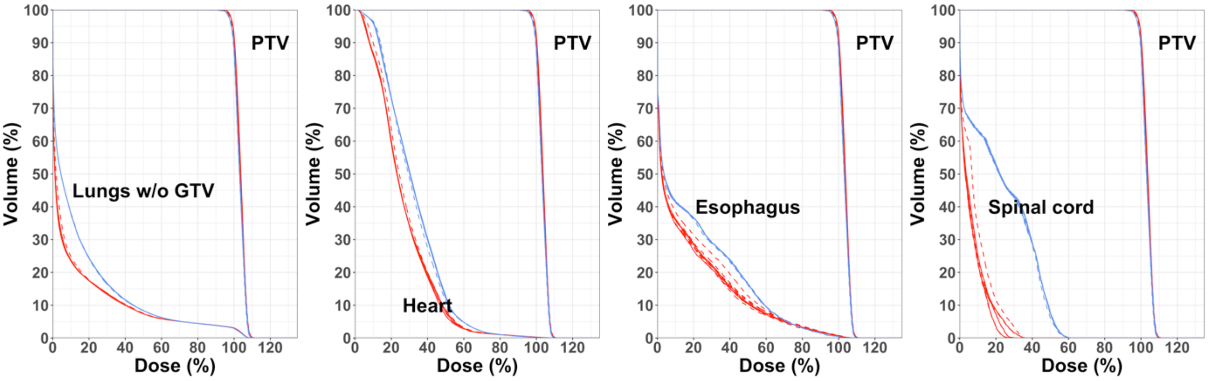

5% over- and undershoot:

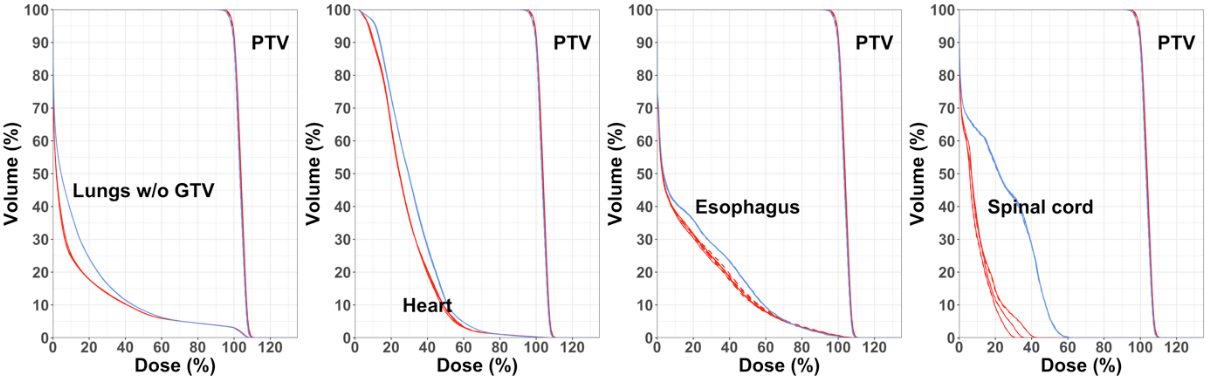

7% over- and undershoot:

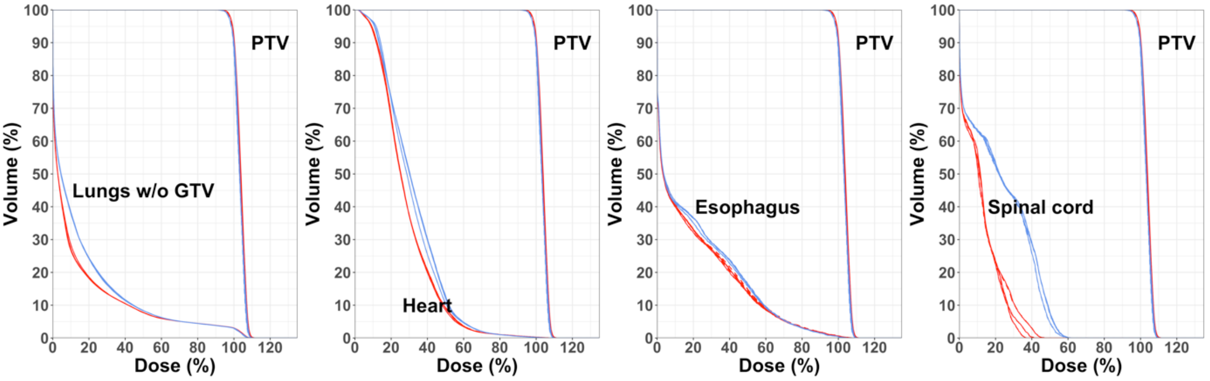

Figure B9: Patient 2 DVHs for IMRT and FHB CPPT scenarios without and with (±3%, ±5% and ±7%) *range robust optimization in the adaptive regime.*

Patient 6 – Non-adaptive regime

No range robust optimization:

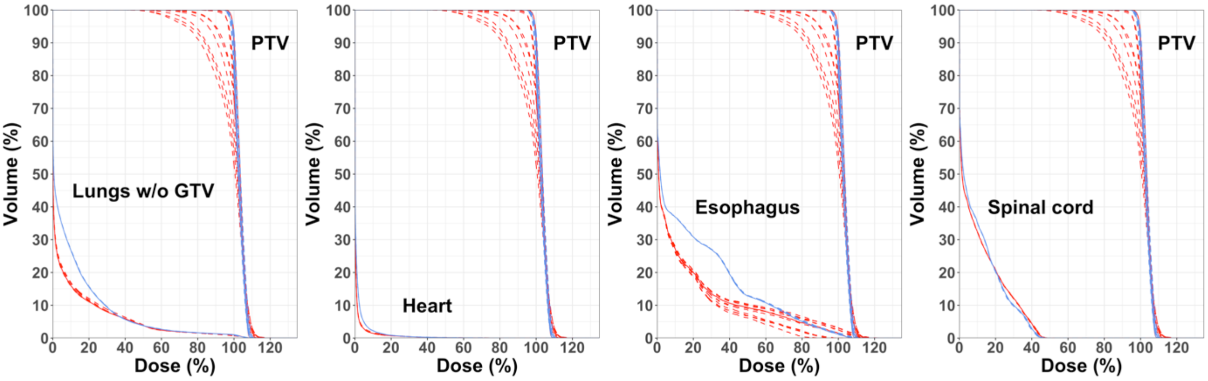

3% over- and undershoot:

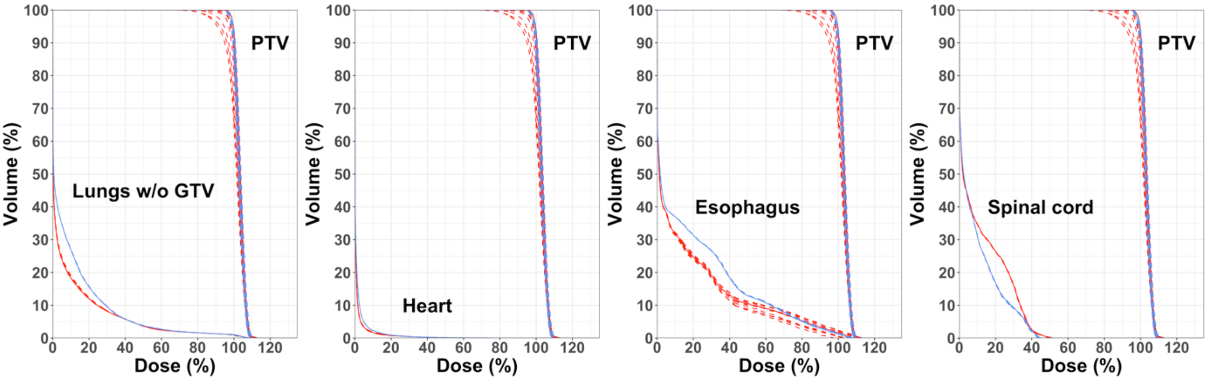

5% over- and undershoot:

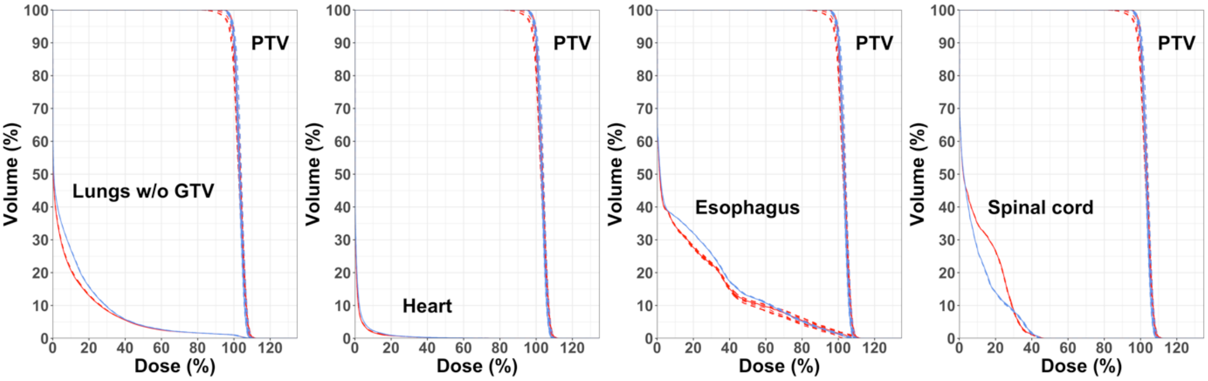

7% over- and undershoot:

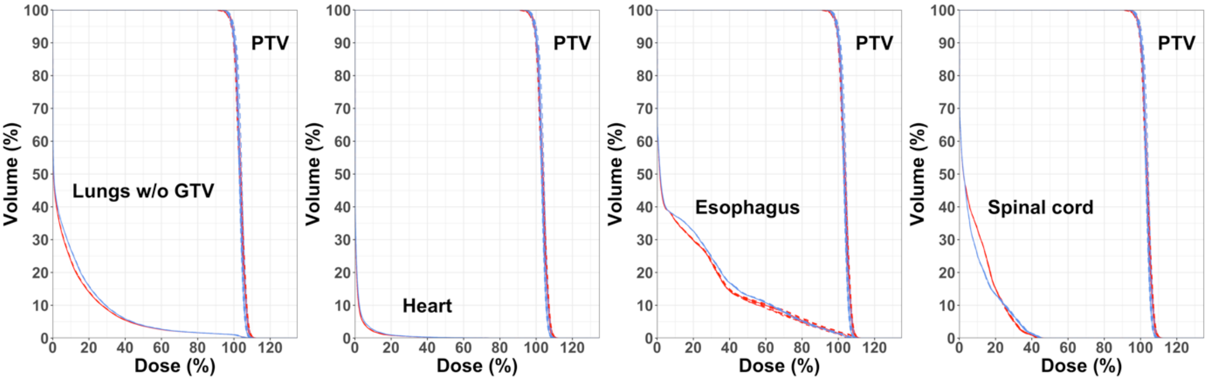

Figure B10: Patient 6 DVHs for IMRT and FHB CPPT scenarios without and with (±3%, ±5% and ±7%) *range robust optimization in the non-adaptive regime.*

Patient 6 – Adaptive regime

No range robust optimization:

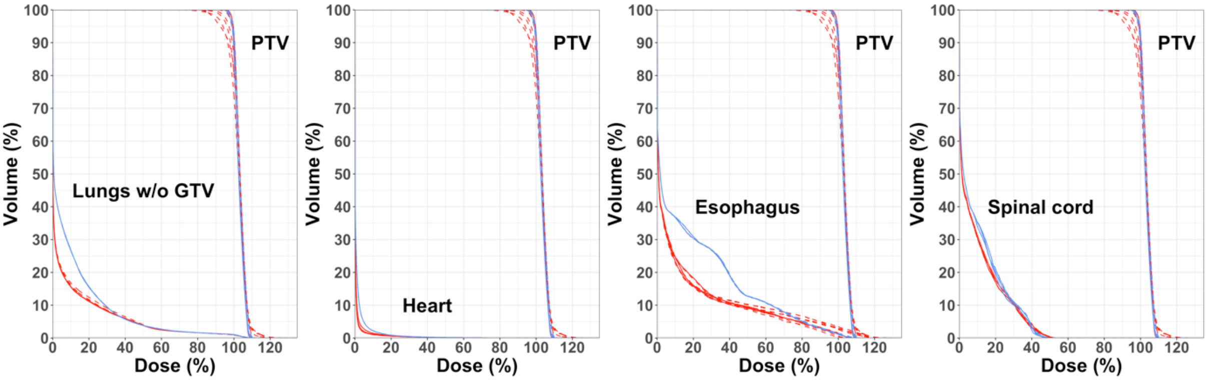

3% over- and undershoot:

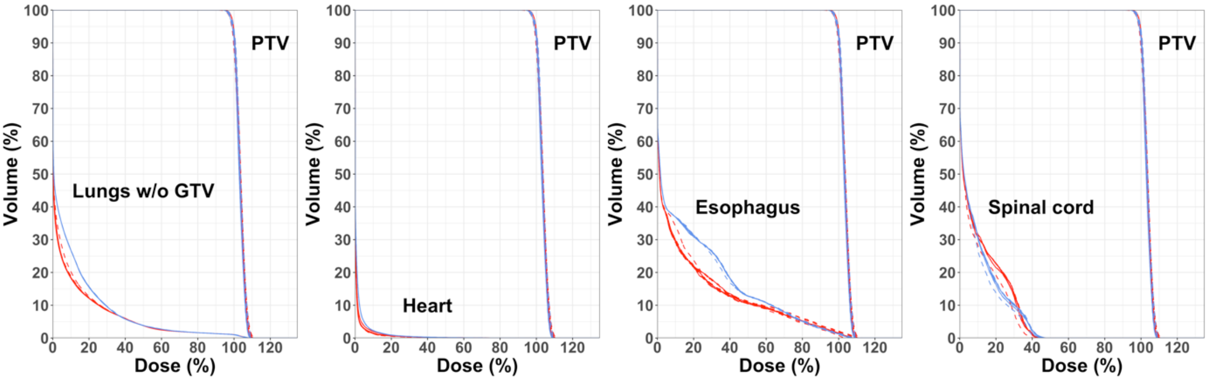

5% over- and undershoot:

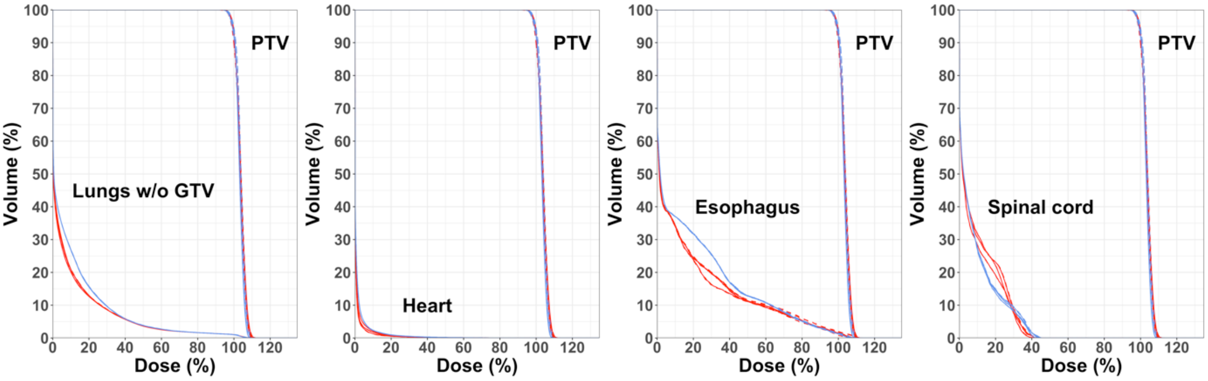

7% over- and undershoot:

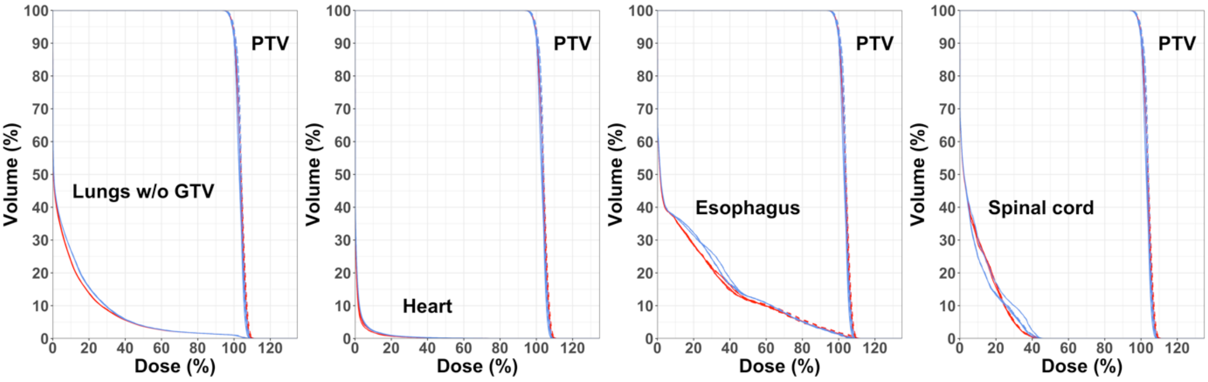

Figure B11: Patient 6 DVHs for IMRT and FHB CPPT scenarios without and with (±3%, ±5% and ±7%) *range robust optimization in the adaptive regime.*

Radiation Pneumonitis	Patient	IMRT	FHB CPPT	Red. to IMRT	Gantry CPPT	Red. To IMRT	Gantry IMPT	Red. To IMRT	FHB IMPT	Red. To IMRT
	P1	14.0	8.2	-5.9	9.0	-5.0	8.5	-5.6	8.1	-5.9
	P2	10.5	6.4	-4.0	6.9	-3.6	4.4	-6.1	5.8	-4.7
	P3	8.7	5.3	-3.4	5.0	-3.8	4.4	-4.3	4.9	-3.8
	P4	8.9	6.7	-2.2	5.9	-3.0	5.2	-3.7	8.1	-0.8
	P5	27.4	11.3	-16.2	10.6	-16.8	9.7	-17.7	7.7	-19.8
	P6	4.9	3.6	-1.3	3.2	-1.8	2.9	-2.0	3.2	-1.7
	P7	7.3	4.8	-2.5	4.6	-2.7	5.2	-2.1	5.9	-1.5
	**Avg**	**11.7**	**6.6**	**-5.1**	**6.5**	**-5.2**	**5.7**	**-5.9**	**6.2**	**-5.4**

2-year mortality	Patient	IMRT	FHB CPPT	Red. to IMRT	Gantry CPPT	Red. To IMRT	Gantry IMPT	Red. To IMRT	FHB IMPT	Red. To IMRT
	P1	47.6	45.3	-2.3	44.0	-3.6	44.1	-3.4	44.1	-3.5
	P2	58.7	54.8	-3.9	47.6	-11.1	43.2	-15.5	37.2	-21.5
	P3	35.4	34.9	-0.6	32.8	-2.6	31.4	-4.0	30.7	-4.8
	P4	60.7	59.3	-1.4	59.1	-1.6	57.1	-3.6	57.2	-3.5
	P5	78.5	72.2	-6.3	72.5	-5.9	71.8	-6.7	70.0	-8.5
	P6	31.4	30.6	-0.9	29.4	-2.1	29.0	-2.4	29.5	-2.0
	P7	47.2	45.2	-2.0	43.3	-3.9	36.6	-10.6	36.9	-10.2
	**Avg**	**51.4**	**48.9**	**-2.5**	**47.0**	**-4.4**	**44.7**	**-6.6**	**43.7**	**-7.7**

Esophageal toxicity	Patient	IMRT	FHB CPPT	Red. to IMRT	Gantry CPPT	Red. To IMRT	Gantry IMPT	Red. To IMRT	FHB IMPT	Red. To IMRT
	P1	32.5	26.6	-5.9	21.2	-11.3	14.8	-17.7	31.3	-1.1
	P2	29.8	27.2	-2.6	16.9	-13.0	10.0	-19.8	29.7	-0.1
	P3	46.2	40.7	-5.5	37.8	-8.4	32.3	-14.0	26.8	-19.4
	P4	9.0	6.8	-2.2	5.6	-3.4	4.4	-4.6	6.2	-2.8
	P5	54.3	46.6	-7.7	37.2	-17.1	29.0	-25.3	40.2	-14.2
	P6	26.3	16.1	-10.3	10.5	-15.9	8.5	-17.9	10.4	-16.0
	P7	52.7	50.3	-2.4	50.7	-2.0	44.9	-7.7	45.3	-7.3
	**Avg**	**35.8**	**30.6**	**-5.2**	**25.7**	**-10.2**	**20.6**	**-15.3**	**27.1**	**-8.7**

Table B5: NTCP values for the non-adaptive regime the values for each patient are the average over CT0-CT9.

Radiation Pneumonitis	Patient	IMRT	FHB CPPT	Red. to IMRT	Gantry CPPT	Red. To IMRT	Gantry IMPT	Red. To IMRT	FHB IMPT	Red. To IMRT
	P1	13.6	7.5	-6.1	8.1	-5.5	7.1	-6.5	7.2	-6.4
	P2	10.0	6.3	-3.7	6.3	-3.7	4.1	-5.9	5.6	-4.4
	P3	8.8	5.2	-3.6	4.8	-4.0	4.4	-4.4	4.8	-3.9
	P4	8.6	6.3	-2.3	5.8	-2.8	5.4	-3.2	7.3	-1.3
	P5	27.1	9.1	-18.1	9.4	-17.7	8.7	-18.5	6.1	-21.1
	P6	4.9	3.6	-1.3	3.2	-1.7	2.9	-2.0	3.2	-1.7
	P7	7.3	4.7	-2.6	4.6	-2.7	5.2	-2.1	5.8	-1.5
	**Avg**	**11.5**	**6.1**	**-5.4**	**6.0**	**-5.4**	**5.4**	**-6.1**	**5.7**	**-5.8**

2-year mortality	Patient	IMRT	FHB CPPT	Red. to IMRT	Gantry CPPT	Red. To IMRT	Gantry IMPT	Red. To IMRT	FHB IMPT	Red. To IMRT
	P1	47.5	44.6	-2.9	44.0	-3.6	43.8	-3.7	42.4	-5.2
	P2	58.4	54.7	-3.7	44.7	-13.7	38.8	-19.7	35.8	-22.6
	P3	35.4	34.7	-0.8	32.7	-2.8	31.6	-3.8	30.7	-4.8
	P4	60.4	58.7	-1.7	58.3	-2.1	55.9	-4.5	56.0	-4.4
	P5	78.7	72.8	-6.0	72.3	-6.4	71.6	-7.1	71.3	-7.5
	P6	31.4	30.2	-1.3	29.4	-2.0	29.1	-2.3	29.3	-2.1
	P7	47.1	45.0	-2.2	43.2	-3.9	36.6	-10.5	36.9	-10.2
	**Avg**	**51.3**	**48.7**	**-2.6**	**46.4**	**-4.9**	**43.9**	**-7.4**	**43.2**	**-8.1**

Esophageal toxicity	Patient	IMRT	FHB CPPT	Red. to IMRT	Gantry CPPT	Red. To IMRT	Gantry IMPT	Red. To IMRT	FHB IMPT	Red. To IMRT
	P1	31.9	20.3	-11.6	19.3	-12.6	12.1	-19.9	18.5	-13.5
	P2	29.2	20.3	-8.9	12.3	-17.0	6.1	-23.1	17.2	-12.0
	P3	46.2	42.3	-3.9	38.0	-8.2	33.5	-12.7	29.8	-16.4
	P4	8.7	6.4	-2.3	5.3	-3.4	4.3	-4.4	5.7	-3.0
	P5	53.9	44.7	-9.2	42.1	-11.9	35.3	-18.6	36.3	-17.6
	P6	26.0	16.4	-9.6	11.8	-14.2	10.3	-15.7	12.4	-13.6
	P7	52.6	51.2	-1.4	51.3	-1.3	45.7	-6.9	48.7	-3.9
	**Avg**	**35.5**	**28.8**	**-6.7**	**25.7**	**-9.8**	**21.0**	**-14.5**	**24.1**	**-11.4**

Table B6: NTCP values for the adaptive regime the values for each patient are the average over CT1-CT9.

Supplement C

In the following, the difficulty of finding the optimal beam angles is discussed using the example of Patient 4. In our work, we used beam configuration 1 (200°, 240°, 340°) which was used for this patient in a previously published work[30] and is shown in Figure C1 A. The rational for these beam angles were to provide clinically adequate dose across the PTV, to spare the contralateral lung completely and to help plan robustness. On the other hand, one could argue that these angles are not optimal in terms of lung sparing. As an example, a treatment plan using a second beam configuration 2 (295°, 325°, 355°) omitting the posterior beams and trying to reduce the proton path through lung was optimized for Patient 4 (Figure C1 B). In Figure C1 C, the DVHs of the two beam configurations are shown for the lungs and the PTV. Beam configuration 2 can indeed reduce the low and medium doses to the lungs which leads to a reduction of the NTCP for radiation pneumonitis of 1.7% (Figure C1 D). Also, the NTCP for esophageal toxicity reduces by 1.3%. However, opposing these benefits, it can be seen in the DVH, that the treatment plan quality of the PTV is reduced. Furthermore, the NTCP for the most severe side effect, the 2-year mortality, is increased by 1.7%. These opposing benefits make it extremely difficult to judge on the optimum of beam angle choice in regards of all possible treatment goals, which was not the topic of this work.

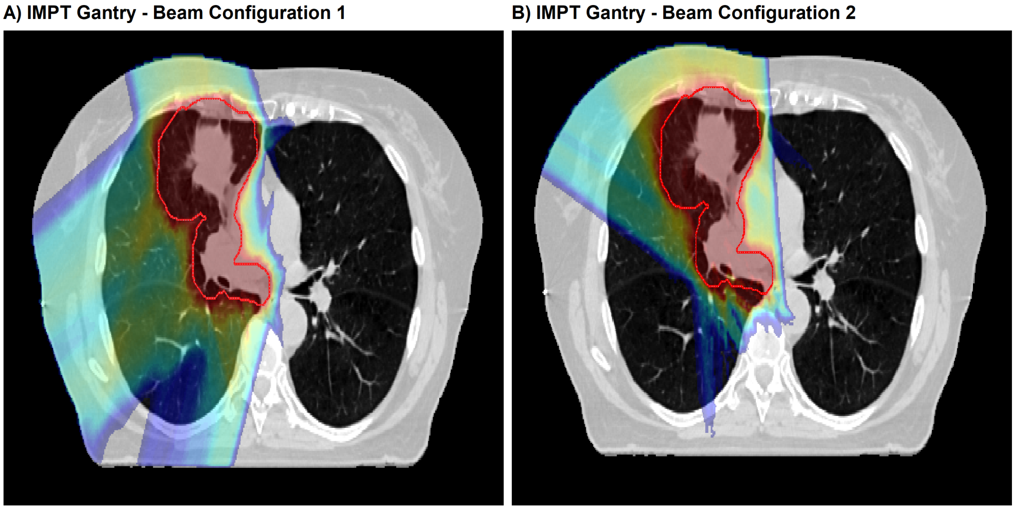

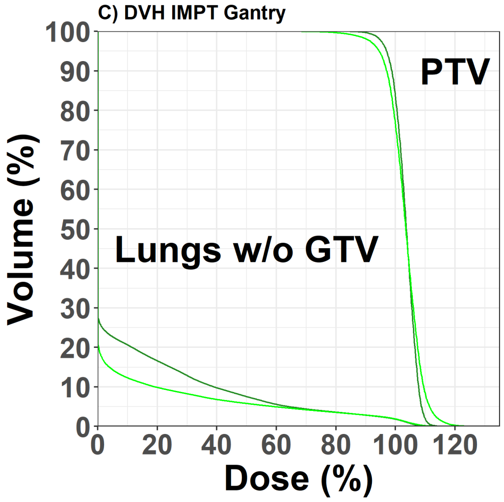

D) NTCP [%]

	Beam Config. 1	Beam config. 2
Radiation Pneumonitis	5.3	3.6
2-year mortality	56.1	57.8
Esophageal Toxicity	4.3	3

Figure C1: A) & B) IMPT gantry dose distributions for Patient 4 using two different beam configurations. C) DVHs for the PTV and the lungs without GTV for beam configuration 1 (dark green) and beam configuration 2 (light green). D) NTCP values for the three investigated side effects for each beam configuration.
